# Bright and Photostable Fluorescent Metal Nanocluster Supraparticles from Invert Emulsions

**DOI:** 10.1002/anie.202210808

**Published:** 2022-08-31

**Authors:** Shaochen Zhou, Bo Peng, Yanyan Duan, Kai Liu, Olli Ikkala, Robin H. A. Ras

**Affiliations:** ^1^ Department of Applied Physics School of Science Aalto University 00076 Espoo Finland; ^2^ IMDEA Materials Institute Calle Eric Kandel 2 28906 Getafe Spain; ^3^ Department of Bioproducts and Biosystems School of Chemical Engineering Aalto University 00076 Espoo Finland

**Keywords:** Light-Emitting Diode, Metal Nanoclusters, Photoluminescence, Photostability, Supraparticles

## Abstract

Fluorescent supraparticles of gold, silver and copper nanoclusters are synthesized by simply drying of invert emulsions, resulting in a dozen‐fold increase in photoluminescence quantum yield (up to ≈80 %) and a significant improvement in photostability. The inhibition of the ligand twisting during the intramolecular charge transfer is found to be responsible for the enhancement, especially for the gold nanocluster supraparticles. This research provides a general, flexible, and easy method for producing highly luminescent and photostable metal nanocluster‐based materials that promise practical applications in white‐light‐emitting diodes.

## Graphical abstract

Supraparticles of gold, silver and copper nanoclusters are formed by drying the water droplets in invert emulsions, resulting in a profoundly enhanced photoluminescence emission and resistance to photobleaching. The supraparticle structure greatly enhances the performance, where the inhibition of the twisted intermolecular charge transfer is one of the responsible causes.

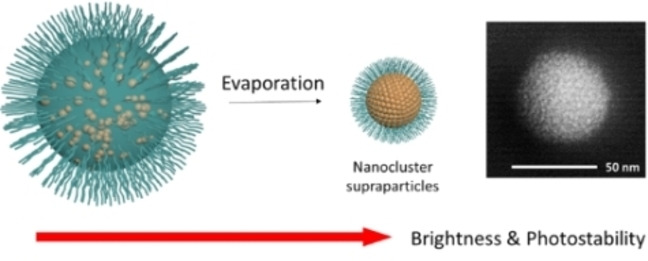

Fluorescent metal nanoclusters (MNCs), composed of several to tens of metal atoms, emerge as promising materials in various applications such as bio/chemo‐analysis, catalysis, biomedicine, etc.[^1^, ^2^] Nevertheless, MNCs typically suffer from low photoluminescence quantum yields (PLQYs) and poor photostability that hinder their applications. Research efforts in the past decades have directed attention to enhancing the PLQYs of MNCs. Assembling MNCs to trigger the aggregation‐induced emission enhancement (AIEE) effect is among the most effective strategies for high PLQYs.[^3^, ^4^] Bright assemblies of MNCs have been obtained through solvent induction,[^5^, ^6^] gelation,^[7]^ intermolecular interaction,[^8^, ^9^] and so forth. However, the resistance of MNCs to photobleaching, namely photostability, has been overlooked. Conventional methods to improve the photostability of fluorescent materials like encapsulation in matrixes can decrease PLQYs.^[10]^ Therefore, the challenge to improve simultaneously the emission brightness and photostability of MNCs remains.

The emulsion techniques offer advantages in manipulating the PL properties of MNC assemblies.^[11]^ Here, we introduce the formation of nanocluster supraparticles from invert emulsions, as a general, straightforward, and effective strategy to achieve improvement in performance. Highly luminescent and photostable gold, silver, and copper nanocluster supraparticles (SPs) can be easily produced by constraining MNCs within water‐in‐oil emulsions and drying water droplets gradually, avoiding complicated molecular engineering. Remarkably, supraparticles of gold nanoclusters can reach a PLQY of ≈80 %, and those of silver nanoclusters exhibit a nearly non‐photobleachable feature. The supraparticle structure plays a crucial role for the significant performance enhancement, as it curbs PL‐unfavorable motions. Notably, the twisted intramolecular charge transfer within supraparticles in contrast to discrete nanoclusters can be considerably suppressed, as also verified by density functional theory.

The fluorescent gold nanoclusters (AuNCs) synthesized by adapting the reported procedures,^[12]^ are selected as the model MNCs in this work (Figure S1). As shown in Figure 1a, the hydrophilic AuNCs are caged in invert emulsions, where surfactants stabilize water droplets in the oil phase. The diffusion tendency of particles in the drying reverse micelles can be studied by the Peclet number: $Pe=\frac{R^{2}}{Dt}\;$ ,^[13]^ where *Pe* stands for the Peclet number, *R* refers to the radius of water droplets, *t* the evaporation time, and *D* the diffusion coefficient of the AuNCs in water. The *Pe* number is calculated to be 1.4×10^−6^–3.9×10^−5^, indicating that particles diffuse uniformly within the water droplets instead of accumulating at the interface when the oil‐water boundary is shrinking. Upon the confinement by the water droplets, AuNCs gradually cluster together to generate gold nanocluster supraparticles, denoted as AuNC‐SPs, driven by the entropy maximization.^[14]^

**Figure 1:**
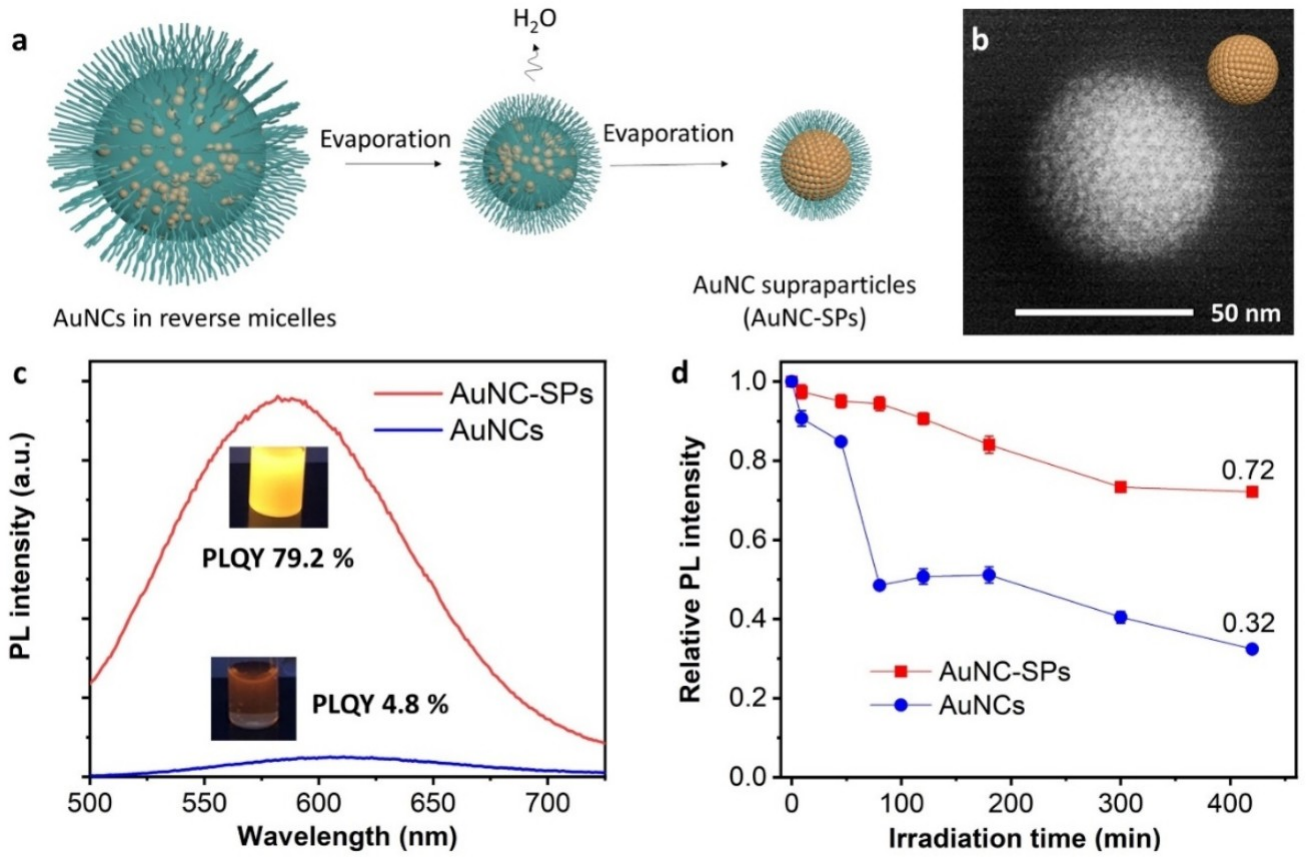
a) Schematic formation process of AuNC‐SPs. b) Scanning transmission electron microscopy image of AuNC‐SPs. c) PL spectra of AuNCs and AuNC‐SPs. d) Relative PL intensity evolution of AuNCs and AuNC‐SPs under continuous UV irradiation at 365 nm, 5.3 mW cm^−2^.

Figure 1b shows the micromorphology of the AuNC‐SPs: well‐defined spherical assemblies of AuNCs as verified by the elemental analysis (Figures S2–S4). The spherical morphology complies with the interfacial tension minimization of the oil‐water boundary. Surprisingly, the AuNC‐SPs show a considerably stronger emission than that from discrete AuNCs (Figure 1c). Both PL emission spectra and the photograph verify the brightness enhancement. Notably, the integrated PL intensity of AuNC‐SPs is tunable by varying the synthetic conditions, achieving 20–60 times that of AuNCs (Figure S6). Specifically, the PLQY of AuNC‐SPs reaches 79.2 % from 4.8 % of AuNCs (Figure 1c). Also, the photostability of AuNC‐SPs is superior (Figure 1d). The emission intensity of AuNCs decreases by ≈68 % after 420‐minute UV exposure at 365 nm, 5.3 mW cm^−2^, while that of AuNC‐SPs is only ≈28 % after the same treatment, suggesting a better resistance to photobleaching.

The luminescence of AuNC‐SPs arises from AuNCs, but is significantly enhanced. The PL of AuNCs stems from the Au^I^‐glutathione thiolate oligomers supported by the Au^0^ clusters,^[12]^ which undergoes an intramolecular charge transfer (ICT) between the ligands and gold.^[15]^ The twist of the electron donors (the ligands) could occur during the ICT process (Figures 2a,b), which is known as the twisted intramolecular charge transfer (TICT), one major cause to decrease the PLQY and photostability of many fluorophores.[^16^, ^17^]

**Figure 2:**
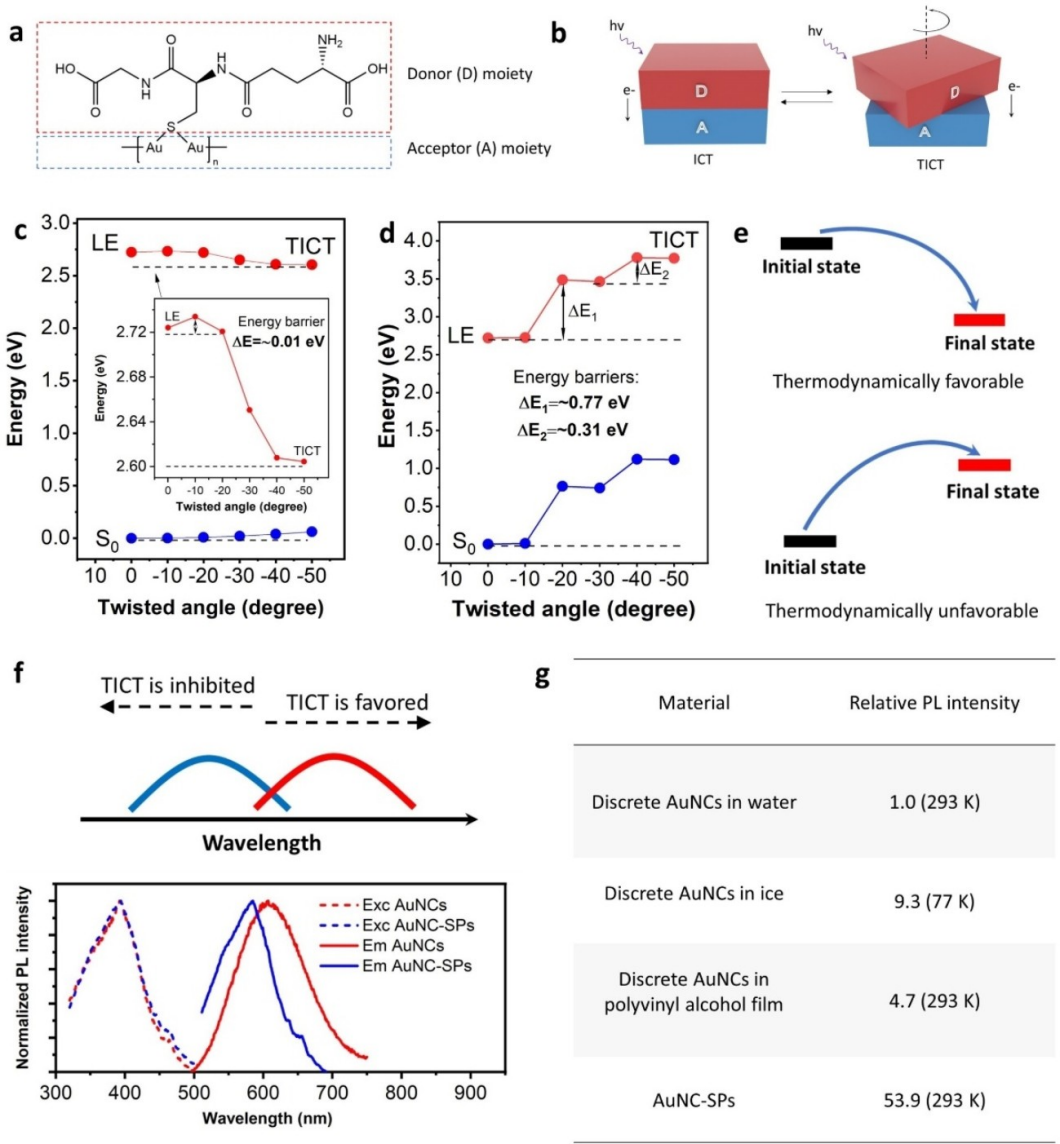
The mechanism behind the performance enhancement of AuNCs in supraparticles. a) The electron donor and acceptor moieties of Au^I^‐SG thiolate oligomers, which are on the surface of AuNCs. b) The ICT and TICT processes upon light excitation. c,d) Energy potentials of the ground states (blue) and first excited states (red) in the emissive Au^I^‐glutathione thiolate oligomer, as a function of rotation angle of the glutathione ligand. The emissive Au^I^‐glutathione thiolate oligomer is from the discrete AuNCs in water (c), and aggregated AuNCs in supraparticles (d), respectively. e) Theoretical modes that indicate thermally favorable and unfavorable processes. f) The presence of TICT red shifts the PL emission (up). The normalized excitation (dash line) and emission (solid line) spectra of both AuNCs and AuNC‐SPs (down). The emission peak blue shifts in AuNC‐SPs due to TICT inhibition. g) Relative PL intensity of AuNCs in water (reference), frozen ice, polyvinyl alcohol film and supraparticles.

Time‐dependent density functional theory calculations reveal that individual AuNCs in water are prone to TICT (Figure 2c). The energy barrier is close to zero (≈0.01 eV) between the locally excited (LE) and the TICT states. The TICT state is located at a lower energy potential than the LE state. The results indicate that the twist of the ligands is highly favorable upon excitation (Figure 2c). The high TICT tendency explains the inferior PLQY and photostability of AuNCs in water. For the AuNCs within the supraparticle structure, the energy barrier for ligand twisting is high and increases upon further twisting in the excitation state, unraveling the twist of ligands is extremely unfavorable (Figures 2d,e). In addition, the presence of TICT can decrease the emission energy profile by diminishing the energy gap between the excited and ground states (Figure 2d). It explains why the peak wavelength of AuNCs is longer than that of AuNC‐SPs (Figure 2f). All results demonstrate the efficient TICT restriction in AuNC‐SPs.

The rigid supraparticle structure, as a result of closely packed nanoclusters, leads to the effective TICT suppression, being responsible for the PLQY and photostability enhancement. Other PL‐unfavorable thermal motions, such as ligand fluctuation,[^18^, ^19^] are also physically inhibited in the rigid structure, contributing to the emission enhancement. Besides the supraparticle structure, water plays an important role in the PL properties of AuNC‐SPs. On the one hand, it can easily soften the supraparticle structure, facilitating TICT and resulting in a great PL intensity decrease (Figures S9 and S10); on the other hand, a trace amount of water remained in the supraparticles stabilizes the supraparticle structure (Figure S12) and facilitates the emission enhancement, as evidenced by the PL intensity decrease upon excessive water removal in the supraparticles (Figure S11).

It is well established that the PLQY of MNCs is determined by the rates of irradiative and non‐irradiative transitions: $PLQY=\frac{K_{r}}{K_{r}+K_{nr}}\;$ ^[18]^ where $K_{r}$ and $K_{nr}$ are the rate coefficients of radiative and nonradiative transitions, respectively. Maximizing the radiative fraction is essential for a great PLQY increase, which is achieved by the interplay of the closely packed nanoclusters and the trace amount of water in the supraparticle structure. The interplay can be further validated by the comparison among AuNCs in different matrices (Figure 2g). A 3–9 times increase in emission intensity is observed when the AuNCs are confined in the ice (77 K) or the polymer film (293 K), while the largest increase (≈53 times) is reached in supraparticles, even at a relatively higher temperature (293 K).

The strategy for aggregation‐induced enhancement is also versatile for glutathione‐stabilized silver nanoclusters (AgNCs) and copper nanoclusters (CuNCs). Similar to AuNCs, the PL of discrete AgNCs and CuNCs originates from the ligand‐metal charge transfer.[^20^, ^21^] This means unfavorable TICT can be involved and decrease their PLQY and photostability. By contrast, the clusterization of AgNCs and CuNCs significantly boosts their PLQYs by 22 and 5.5 times, respectively (Figures 3a,c). Photostability of these MNC‐SPs is also improved conspicuously (Figures 3b,c). Especially for AgNC‐SPs, the PL intensity has experienced a slight drop by ≈5 % after 120‐minute UV irradiation. Performance enhancement of these less noble MNC‐SPs yields enormous practical significance, given the greater element abundance and lower cost than their noble counterparts.

**Figure 3:**
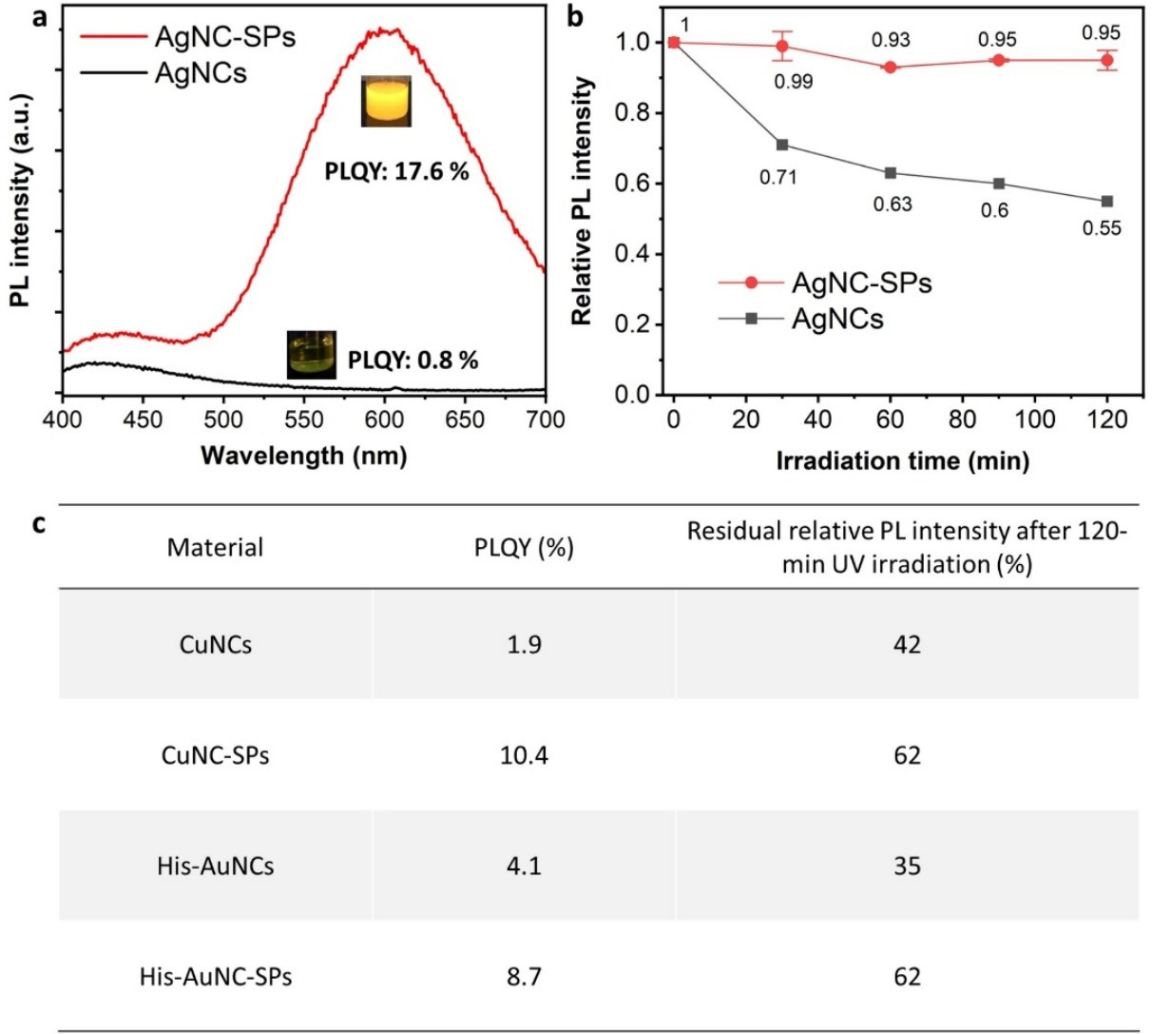
a) PL spectra of AgNCs and AgNC‐SPs. b) Relative PL intensity evolution of AgNCs and AgNC‐SPs under continuous UV irradiation at 365 nm, 5.3 mW cm^−2^. c) The PLQYs and residual relative PL intensities after 120‐minute UV irradiation (365 nm, 5.3 mW cm^−2^) of CuNCs, CuNC‐SPs, His‐AuNCs and His‐AuNC‐SPs.

In addition to glutathione‐stabilized MNCs, the performance enhancement has also been observed in the supraparticles of MNCs that are capped by other ligands and have a different PL origin. The fluorescence of histidine‐stabilized gold nanoclusters (His‐AuNCs), for example, is reported to be less associated with the surface ligand‐metal charge transfer but more with the metal kernel.^[22]^ His‐AuNC‐SPs show enhanced emission and photostability as compared to discrete His‐AuNCs (Figures 3c). The PLQY increase is limited (≈2 folds), largely because the TICT does not affect the PL properties as much as it does in the glutathione‐stabilized MNCs.

In general, formation of the supraparticles from reverse micelles can boost the optical performance of various water‐soluble MNCs as they comply with the following criteria: (a) the Peclet number should be less than 1. As such, the MNCs form supraparticles after drying water from the reverse micelles. (b) The PL of MNCs stems from a ligand‐metal (donor‐acceptor) charge transfer process, in which TICT can be involved. The supraparticle formation can suppress the TICT effect. (c) Alternatively, the PL of MNCs originates from the metal kernel, but thermal motions of surface ligands or nanoclusters can also affect the emission intensity. The supraparticle structure sterically confines the MNCs and rigidifies their surfaces, facilitating the PLQY increase.^[18]^

The general applicability of the synthetic method enables the preparation of various gold, silver and copper nanocluster SPs with enhanced PL performance. Thanks to the high flexibility and controllability in the synthesis, the emission color of the luminescent SPs can be easily tuned in a wide range. The blend of AuNC‐SPs, AgNC‐SPs, CuNC‐SPs, and His‐AuNC‐SPs (at a mass ratio of 1 : 3 : 5 : 10) gives out a bright white light upon UV excitation, having CIE (International Commission on Illumination) color coordinates of (0.31, 0.32) and a color temperature of 6600 K (Figure 4). Reports have demonstrated the application of MNCs, by combining with other luminescent materials, in the white‐light‐emitting diodes (WLEDs) with different merits.[^23^, ^24^, ^25^] The blend of MNC‐SPs exhibits promise in fabricating WLED devices (the inset in Figure 4b), with enhanced PL properties as compared to discrete MNCs, high tunability potential of light color, and flexibility in device fabrication for desired performances.

**Figure 4:**
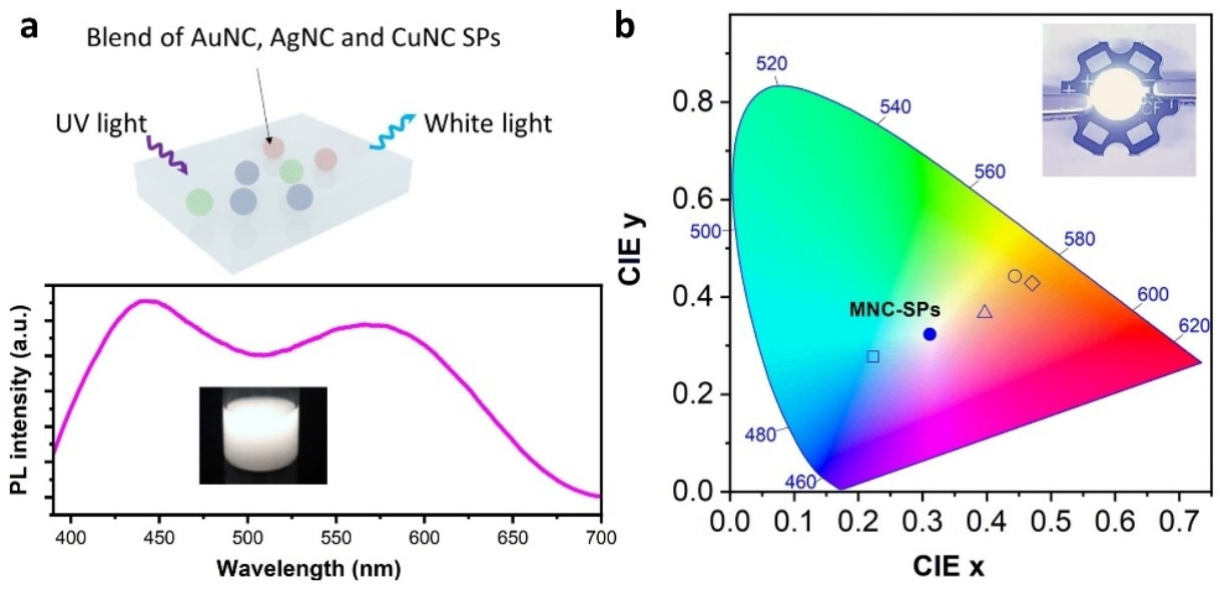
a) White‐light‐emissive blend of AuNC‐SPs, AgNC‐SPs, CuNC‐SPs, and His‐AuNC‐SPs and the PL spectrum. b) The CIE color coordinates of the emissions given out by AuNC‐SPs (hollow circle), AgNC‐SPs (rhombus), CuNC‐SPs (triangle), His‐AuNC‐SPs (square), and the blend of these MNC‐SPs (filled circle). Inset: photograph of a working white‐light‐emitting diode using the blend of the MNC‐SPs as the luminophore.

To summarize, this work provides an easy strategy to prepare bright and photostable supraparticles of gold, silver and copper nanoclusters, in which unfavorable motions, such as the twisted intramolecular charge transfer, are greatly suppressed. The significantly enhanced performance enables a variety of unprecedented applications for the ultrasmall metal nanoclusters.

## Conflict of interest

The authors declare no conflict of interest.

## Supporting information

As a service to our authors and readers, this journal provides supporting information supplied by the authors. Such materials are peer reviewed and may be re‐organized for online delivery, but are not copy‐edited or typeset. Technical support issues arising from supporting information (other than missing files) should be addressed to the authors.

Supporting Information

## Data Availability

The data that support the findings of this study are available from the corresponding author upon reasonable request.
