# Supplementary material for: Bright and Photostable Fluorescent Metal Nanocluster Supraparticles from Invert Emulsions
Source: Angew Chem Int Ed Engl. 2022 Aug 31;61(40):e202210808. doi: 10.1002/anie.202210808 (PMC9804586; doi:10.1002/anie.202210808)
Supplement: Supplementary file 1 — Supporting Information [file ANIE-61-0-s001.pdf]

Angewandte Chemie
Eine Zeitschrift der Gesellschaft Deutscher Chemiker

Supporting Information

# Bright and Photostable Fluorescent Metal Nanocluster Supraparticles from Invert Emulsions

*S. Zhou, B. Peng*, Y. Duan, K. Liu, O. Ikkala, R. H. A. Ras**

# Contents

S1. Materials and methods .......... 2
*Chemicals.* .......... 2
*Preparation of AuNCs* .......... 2
*Synthesis of AuNC-SPs* .......... 2
*Preparation of the AuNC-PVA film* .......... 2
*Preparation of AgNC and AgNC-SPs* .......... 2
*Preparation of CuNCs and CuNC-SPs* .......... 2
*Synthesis of His-AuNCs and His-AuNC-SPs* .......... 3
*Preparation of white light-emitting diodes (LEDs) using MNC-SPs* .......... 3
*Characterization* .......... 3
*Computational calculation* .......... 3
S2. Properties of the GSH-stabilized AuNCs .......... 3
S3. Morphology, structure and composition of AuNC-SPs. .......... 4
S4. The performance tunability of the as-prepared AuNC-SPs .......... 8
*Performance tunability of AuNC-SPs* .......... 9
S5. Computational study: TICT of AuNCs and its restriction in AuNC-SPs. .......... 9
*TICT tendency of AuNCs in water* .......... 9
*TICT tendency of AuNCs in the supraparticle structure* .......... 10
S6. The effect of water on the PL properties of AuNC-SPs .......... 11
*Addition of water into the AuNC-SP system* .......... 11
*Absorption and extraction of water in the AuNC-SP system* .......... 12
*Over-evaporation during the formation of AuNC-SPs* .......... 14
S7. The PL kinetics in the AuNCs .......... 15
S8. PL enhancement of AuNCs in different matrices .......... 16
*PL enhancement of AuNCs in the polymer film* .......... 16
*PL enhancement of AuNCs in the frozen ice* .......... 16
*PL enhancement of AuNCs in the superaparticle structure* .......... 16
S9. Properties of other MNCs and MNC-SPs (M=Au, Ag, and Cu) .......... 17
*Performance enhancement in the supraparticles of AgNCs and CuNCs* .......... 17
*Performance enhancement in the supraparticles of His-AuNCs* .......... 17
*The effect of water in the PL properties of AgNC-SPs and CuNC-SPs* .......... 20
References .......... 21

## S1. Materials and methods

*Chemicals.* Glutathione (GSH, ≥98%), L-histidine (≥99%), gold chloride ($HAuCl_4 \cdot 3H_2O$, 99%), silver nitrite ($AgNO_3$, ≥99.0%), copper nitrite ($Cu(NO_3)_2 \cdot xH_2O$, ≥99.9% trace metals basis), L-ascorbic acid (99%) and the nonionic surfactant, sorbitan monooleate (Span® 80, viscosity 1000-2000 mPa·s), SYLGARD® 184 silicone elastomer kit (polydimethylsiloxane, silicone and curing agent), polyvinyl alcohol (PVA, MW: 13000~25000, 87~89%) are purchased from Sigma Aldrich. Dodecane (99 %) is obtained from Alfa Aesar. All agents are used without further purification. Millipore ultrapure water (18.2 MΩ) is used throughout the work.

*Preparation of AuNCs.* GSH-stabilized gold nanoclusters (AuNCs) are obtained through reported synthetic procedures.[1] In a typical synthesis, 0.5 mL of $HAuCl_4 \cdot 3H_2O$ (20 mM) is added into 4.35 mL of ultrapure water at room temperature (RT). Then, 150 μL of GSH aqueous solution (100 mM) is introduced into the mixture, which is stirred for 15 min until it becomes transparent. The reaction mixture is then heated at 70 °C for 24 h. The as-obtained AuNC solution is purified with dialysis for 12 h, which is then stored at 4 °C for further use.

*Synthesis of AuNC-SPs.* Typically, a two-phase system is obtained by mixing 1 mL AuNCs water solution with 5 mL dodecane, to which 200 mg of the surfactant, Span® 80, is then added. The mixture is stirred at 11000 rpm for 5 min to form a water-in-oil emulsion, which is then stirred at 500 rpm and heated at 80 °C for 2 h in an oil bath, producing AuNC-SPs. Extra surfactants can be removed by centrifugation and redispersion of the precipitants in dodecane or chlorophorm. Different oil/water ratios and surfactant concentration will lead to AuNC-SPs with different PL properties.

*Preparation of the AuNC-PVA film.* 1 mL of AuNCs aqueous solution is mixed with 2 mL of PVA solution (1.5 wt%). The AuNC-PVA film is obtained by water evaporation at 50°C for 24 h.

*Preparation of AgNC and AgNC-SPs.* GSH-stabilized silver nanoclusters (AgNC) are prepared by reported procedures with a slight modification.[2] 300 μL of GSH water solution (50 mM) and 250 μL of $AgNO_3$ solution (20 mM) are mixed with 4.45 mL ultrapure water and stirred at 1100 rpm, RT to prepare Ag(I)-SG complexes. After adjusting pH to 6, the transparent solution is added into a Teflon lining, and then put into an autoclave and heated at 110 °C for 4 h. The as-obtained AgNC solution is purified with dialysis for 6 h, which is then stored at 4°C for further use. In the synthesis of AgNC-SPs, 1 mL of AgNC solution is mixed with 5 mL of dodecane, followed by addition of 200 mg of Span® 80. A water-in-oil emulsion is formed when the mixture is stirred at 11000 rpm for 3 min, which is then stirred at 400 rpm, RT for 72 h. AgNC-SPs are collected by centrifugation under 6000 rpm for 5 min and redispersed in dodecane for subsequent use.

*Preparation of CuNCs and CuNC-SPs.* GSH-stabilized copper nanoclusters (CuNCs) are obtained through a top-down synthetic strategy.[3] Firstly, 1 mL water solution of $CuNO_3$ (100 mM) is added dropwise into 10 mL aqueous solution of ascorbic acid (0.1761 g/mL), which is stirred at 400 rpm, RT for 1 h to produce stable copper nanoparticles. 0.3 mL of the as-prepared copper nanoparticles is added to 3 mL of GSH aqueous solution (50 mM), which is kept under RT for 5 h. Fluorescent CuNCs are prepared through an etching process, which are collected by centrifugation at 11000 rpm for 5 min and dispersed in water. The product is stored at 4 °C for further use. To prepare CuNC-SPs, 1 mL of the CuNCs solution is mixed with 5 mL of dodecane, followed by the addition of 200 mg of Span®80. A microemulsion is formed when the mixture is stirred at 11000 rpm for 2 min, which is then stirred at 400 rpm, RT for 72 h. CuNC-SPs are obtained by centrifugation under 6000 rpm for 5 min and redispersed in dodecane.

*Synthesis of His-AuNCs and His-AuNC-SPs.* Histidine-stabilized gold nanoclusters (His-AuNCs) are obtained following Yang et al's report.[4] 1 ml aqueous solution of $HAuCl_4$ (10 mM) is mixed with 3 mL of histidine solution (100 mM), followed by the incubation at RT for 2 h. An aqueous solution of

$Au_{10}$ clusters is produced and stored at 4 °C for further use. For the synthesis of His-AuNC-SPs, 1 mL of the His-AuNCs solution is mixed with 5 mL of dodecane, followed by addition of 200 mg of Span® 80. A water-in-oil emulsion is formed when the mixture stirred at 11000 rpm for 2 min, which is then stirred at 400 rpm, RT for 72 h. CuNC-SPs are obtained by centrifugation under 6000 rpm for 5 min and redispersed in dodecane for subsequent use.

*Preparation of white light-emitting diodes (LEDs) using MNC-SPs*. The white light-emissive metal nanocluster supraparticles (MNC-SPs) are prepared by mixing 1 mg of CuNC-SPs, 2 mg of His-AuNC-SPs, 0.6 mg AgNC-SPs, and 0.4 mg AuNC-SPs. Then, the MNC-SPs are mixed with 500 mg of SYLGARD® 184 silicone elastomer. The as-synthesized mixture is deposited on commercially available LED chips (3W, $\lambda_{em}$=390 nm) and heated at 50 °C for 12 h. The white-emissive LEDs are herein produced.

*Characterization*. High-resolution transmission electron microscopy (HR-TEM) and scanning transmission electron microscopy (STEM) are performed on JEM-2800 high-throughput analytical TEM (JEOL). The instrument is operated at an acceleration voltage of 200 kV with field-emission guns. Specimens for TEM analysis are prepared by drop-casting colloidal samples in the ultrathin carbon film coated on copper grids. UV-visible absorption spectra are recorded on a UV-Vis-NIR Agilent Cary 5000 spectrophotometer. Dynamic light scattering measurements are performed using Malvern Zetasizer Nano-ZS90. The steady-state PL spectra are obtained from a Quanta Master 40 fluorescence spectrometer. Time-resolved PL spectra and absolute photoluminescence quantum yields (PLQYs) are measured using a FS5 Spectrofluorometer (Edinburgh Instruments). The PL decay curves are collected and analyzed using a time correlation single photon counting (TCSPC) system. PLQYs are obtained using the SC-30 module coupled with an integrating sphere, in which liquid or film samples are excited at 390 nm. Matrix-assisted laser desorption/ionization-time of flight (Maldi-TOF) mass spectroscopy is performed on Bruker Maldi-TOFF D410.

*Computational calculation*. Density functional theory (DFT) and time-dependent (TD)-DFT calculations are carried out with the *Gaussian 16* using the B3LYP function. The Def2TZVP basis set and corresponding effective core potential are used for all gold atoms and the Def2SVP basis set for the rest atoms throughout the calculations.[5,6] Geometry optimizations are performed in the ground state ($S_0$) at first. Then, the molecular structures at the first excited state are optimized based on the optimized molecular structures at the $S_0$ state. Solvent effects are considered using the IEFPCM model. Frequency checks are carried out after each geometry optimization to ensure the minima potential energy surfaces are found and the imaginary frequencies relating to the fixed atoms are ignored.

## S2. Properties of the GSH-stabilized AuNCs

It is well established that the GSH-stabilized AuNCs are Au(0) clusters capped by Au(I)-SG oligomers,[1] as shown in Figure S1a. The Au(I)-SG oligomers are the PL origin of the AuNCs, while the Au(0) kernel, acts as a supporter to stabilize the oligomers on its surface. The Maldi-TOF mass spectrum result indicates that the oligomers are mainly composed of $Au(I)_{12}SG_{13}$, which are supported by Au(0) kernels that are consisted of 3~9 Au(0) atoms. The average PL lifetime is on a microsecond scale, which is 14.9 µs, indicating that the PL is associated with the charge transfer between the ligands and gold atoms.[7]

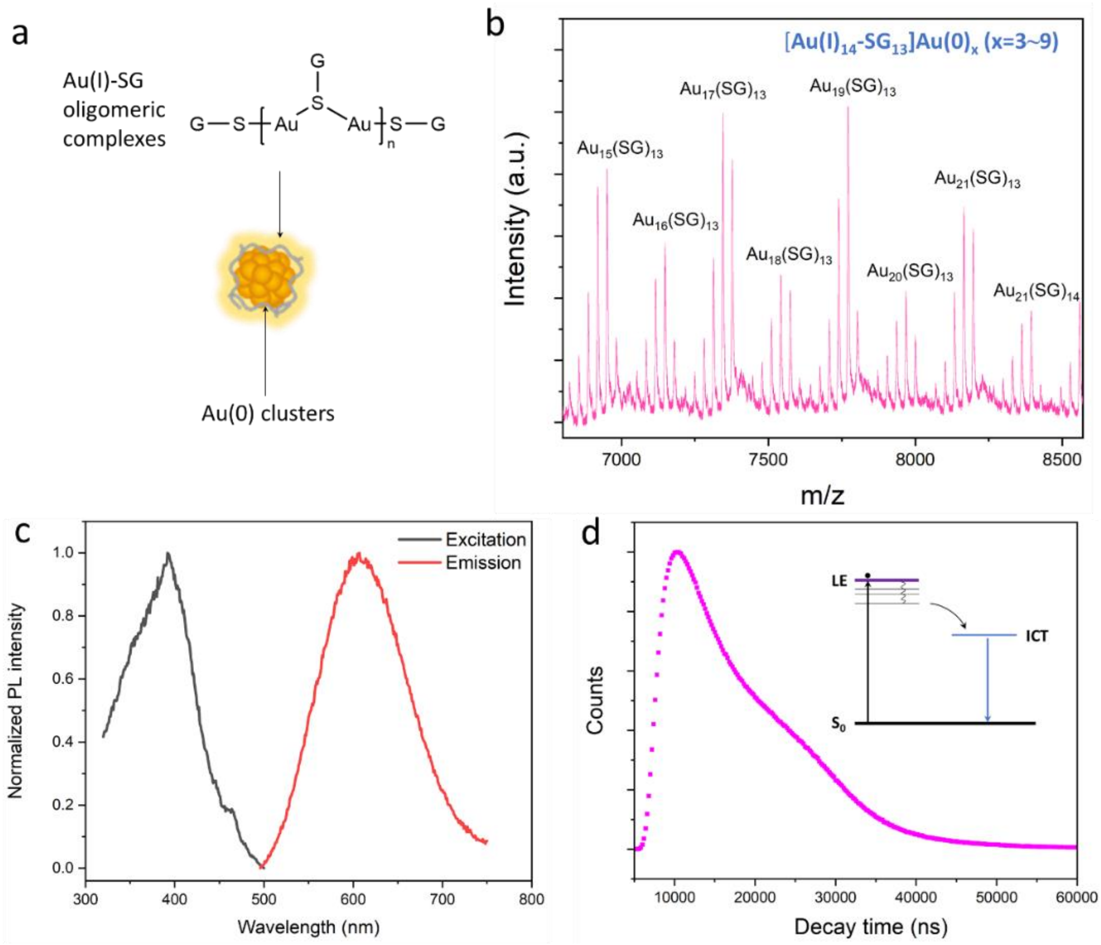


**Figure S1.** Properties of the GSH-stabilized AuNCs. (a) Structure model of the AuNCs. (b) Maldi-TOF mass spectrum of the AuNCs. (c) Normalized excitation and emission spectra of AuNCs. (d) Time-resolved PL spectrum of the AuNCs.

## S3. Morphology, structure and composition of AuNC-SPs.

The STEM images reveal the micromorphology of AuNC-SPs: they are well-defined spherical assemblies of ultrasmall particles (Figure S2), which are assigned to AuNCs as indicated by the EDS results (Figure S4). The spherical structure is caused by the minimization of interfacial tension at the oil-water boundary during the synthetic process. Figures S4c~f exhibit the elemental distribution within the sphere. Two typical elements, Au from the nanoclusters and S from the surface ligands, GSH, are distributed throughout the superstructure, further confirming that they are AuNCs.

Size distributions of AuNCs in the solution and superaparticle are shown in Figure S3. There is no obvious difference in the average particle size, namely ~1.3 nm, ruling out the formation of long-range orderly lattices. Structure and morphology of AuNCs have been retained in the supraparticle.

Figures S2c and S2d show the edge of AuNC-SPs at a higher magnification. A clear distance between the ultrasmall particles can be observed. The average center to center distance of neighboring particles is measured to be ~2.25 nm. Given the average diameter is ~1.33 nm, the gap between neighboring particles is thereby ~0.9 nm.

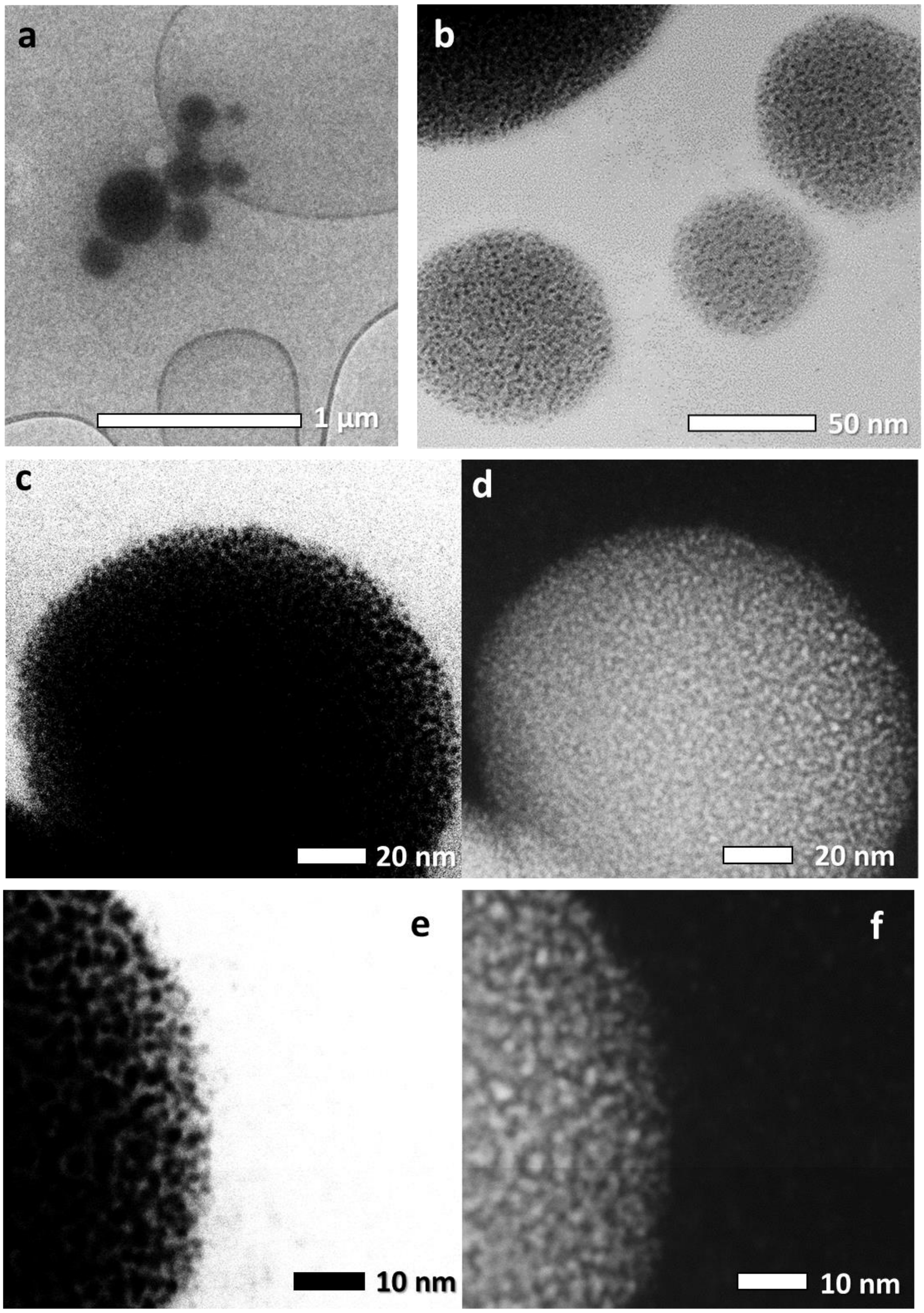


**Figure S2.** TEM images of AuNC-SPs. Supraparticles with a size of 45 nm~300 nm can be observed (a, b). The spherical assemblies are composed of ultrasmall nanoclusters, namely AuNCs (c~f).

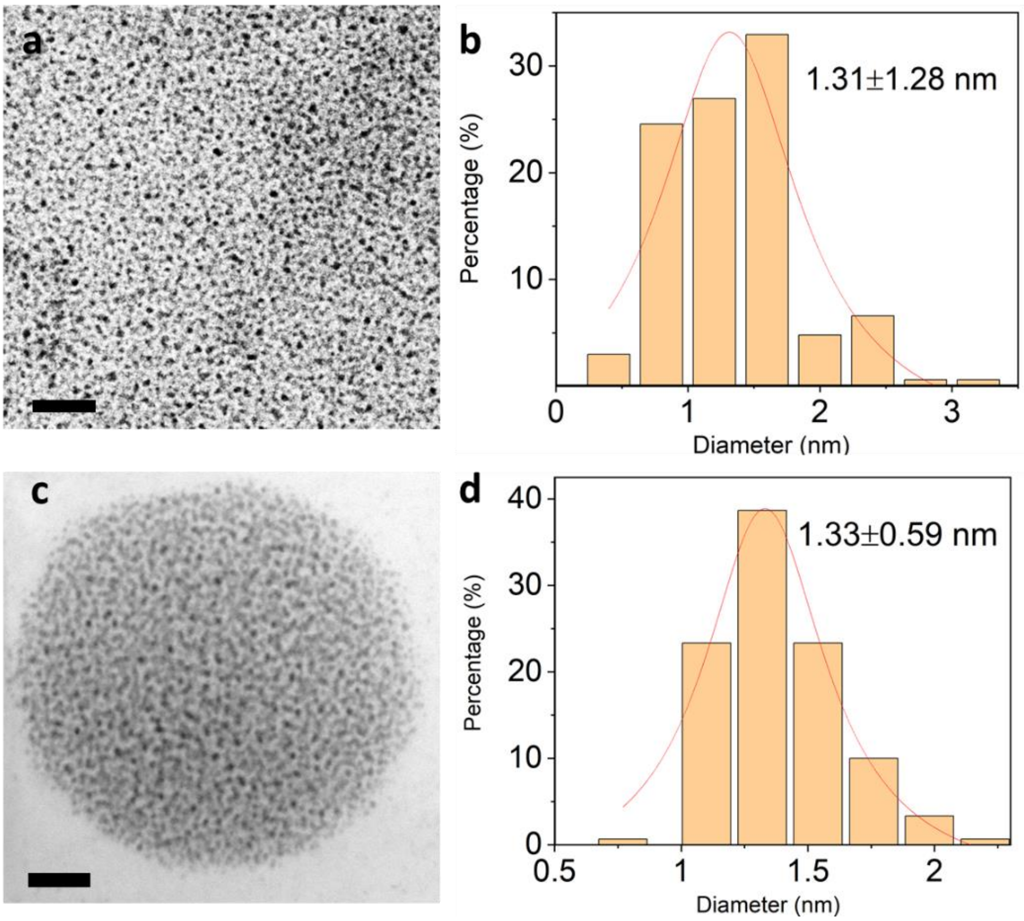


**Figure S3.** Size distributions of AuNCs in discrete (a, b) and supraparticle (c, d) format, respectively. Scale bars: 20 nm.

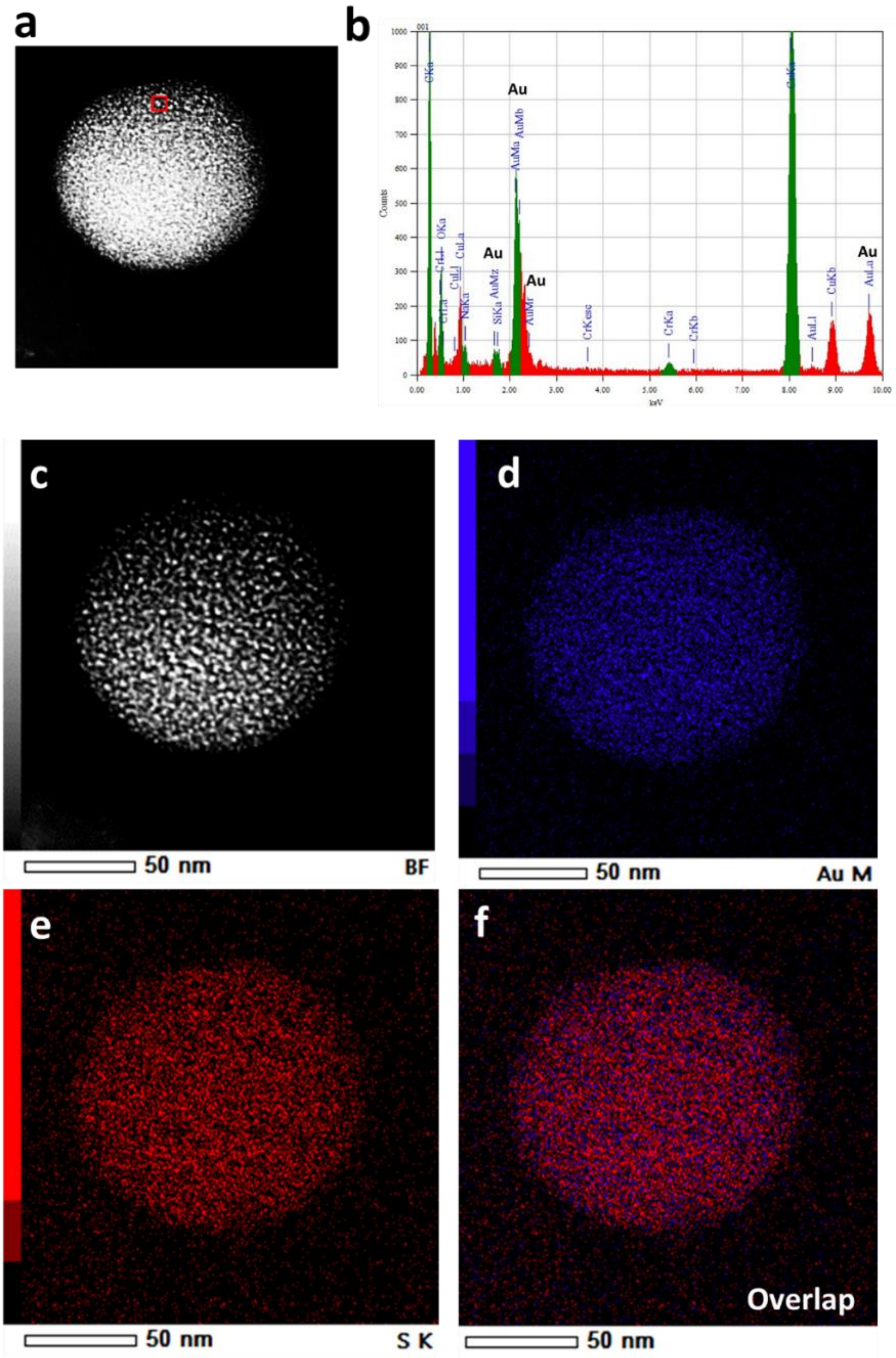


**Figure S4.** EDS spectrum (a, b) and X-ray mapping results (c, d, e, f) of AuNC-SPs. Au and S elements, the main components of AuNCs, are distributed throughout the supraparticle structure.

AuNC-SPs are clusters of AuNCs that are stabilized by the surfactants on their surface. UV-vis absorption spectra of AuNCs, AuNC-SPs and the surfactants, are shown in Figure S5a. The absorption peak at ~390 nm assigned to AuNCs can be observed in the spectrum of AuNC-SPs, where absorption peaks at ~270 and ~281 nm attributed to the surfactant, are also apparently shown, however not in the spectrum of AuNCs. Thus, AuNC-SPs are composed of both AuNCs and surfactants.

The FT-IR results provide additional evidence of the presence of surfactant on AuNC-SPs (Figure S5b). All peaks observed in the spectrum of AuNCs can be found in that of AuNC-SPs, since AuNC-SPs are clusters of AuNCs. Many peaks attributed to the surfactant can be observed in the spectrum of AuNC-SPs as well: peaks at 2936 and 2885 $cm^{-1}$ are assigned to the stretching vibration of -C-H bonds, which mainly come from the carbon chain of the surfactant; and the stretching vibration of -OH bonds from the surfactant have also contributed to the wide transmittance band from 3300 to 3500 $cm^{-1}$. The binding between the surfactants and AuNC-SPs is most likely due to the affinity behavior between the C=O bonds of the surfactant and the Au(I)-SG oligomers on the surface of AuNCs, resulting in the formation of a surfactant shell.

In general, the surfactant shell plays an important role in the performance enhancement for AuNC-SPs. Firstly, the surfactant shell confines the AuNCs within the spherical structure, resulting in a drastic increase in PL intensity. Secondly, the surfactant shell can keep the AuNC-SPs from harmful or destructive species that greatly quench the PL intensity. The quenchers, can neither easily reach their surface nor diffuse into the structure, making the PL intensity decrease rather slowly under long-time irradiation.

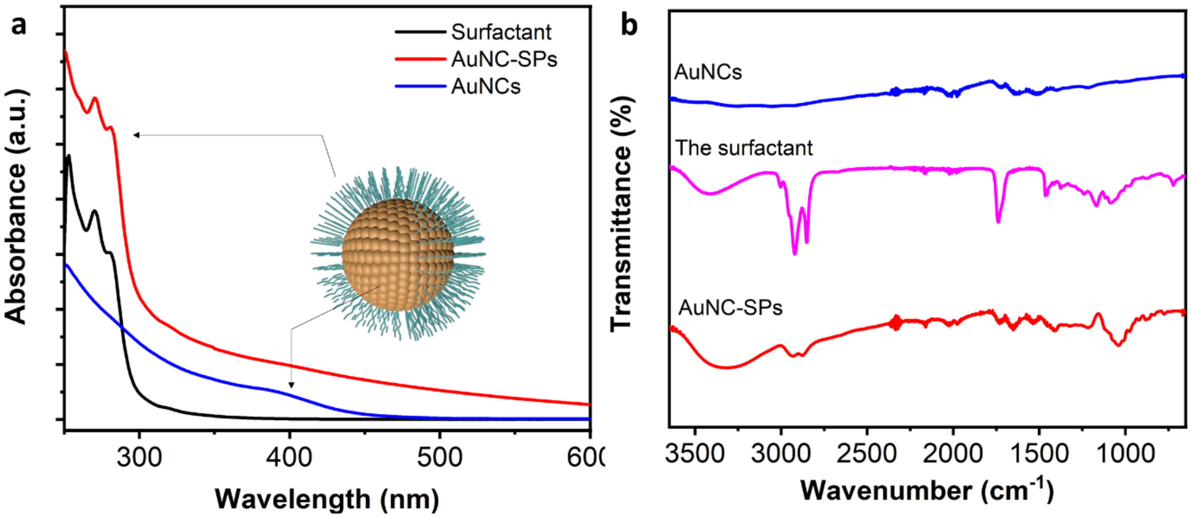


**Figure S5.** UV-vis spectra (a) and FTIR (b) of the AuNCs, AuNC-SPs, and the surfactant, Span-80. Spectral results indicate the supraparticles are composed of AuNCs and the surfactants.

## S4. The performance tunability of the as-prepared AuNC-SPs

*Performance tunability of AuNC-SPs*. When fixing the oil/water volume ratio at 6:1, for example, surfactant concentration matters in the PL properties of AuNC-SPs. A modest surfactant concentration (0.05~0.37 mmol/mL) can lead to at least a twenty-fold increase in the emission intensity with respect to AuNCs (Figure S7a). PL intensity of AuNC-SPs reaches 54 times as that of AuNCs at the surfactant concentration of 0.09 mol/mL. However, too much surfactant (> 0.77 mol/mL) could bring down the emission enhancement, due to the formation of smaller supraparticles. Surfactant concentration affects the photostability greatly as well (Figure S7b). AuNC-SPs obtained from a low surfactant concentration, 0.05 mmol/mL, show a poorer resistance to photobleaching with emission intensity declining to 35% after 420-min UV irradiation, almost the same with the AuNCs (~32%). But a slightly higher surfactant concentration can substantially improve the photostability. In general, the more surfactant there are on the surface of AuNC-SPs, the more photostable they are. The emission intensity can stay up to ~80% of the initial value after UV irradiation when the concentration reaches 0.36 mmol/mL.

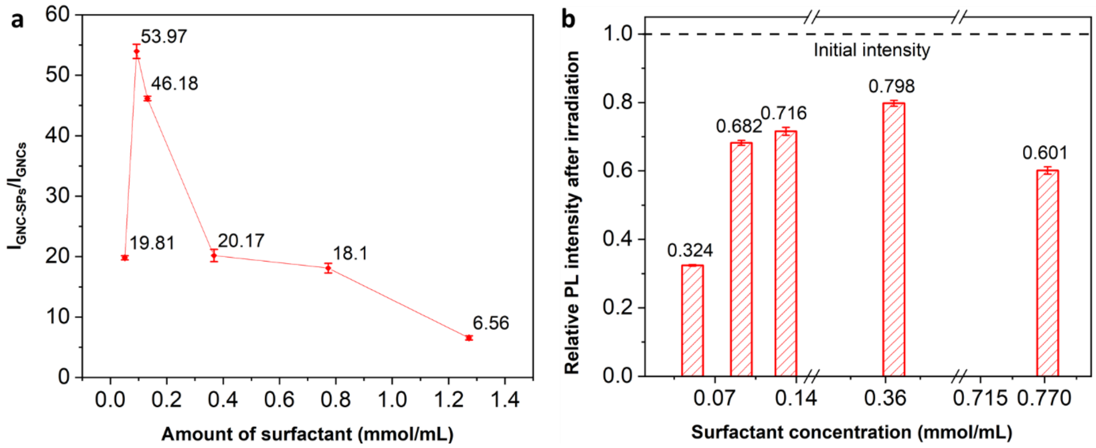


**Figure S6.** Performance tunability of AuNC-SPs. (a) PL intensity ratio of AuNC-SPs versus equivalent amount of AuNCs. AuNC-SPs are obtained at different surfactant concentrations. The higher the concentration, the greater the emission enhancement. (b) Relative PL intensity of AuNC-SPs after 420-min continuous UV irradiation at 365 nm, 5.3 mW/cm$^2$. AuNC-SPs are obtained at different surfactant concentrations. The higher the relative intensity, the better the resistance to photobleaching.

## S5. Computational study: TICT of AuNCs and its restriction in AuNC-SPs.

*TICT tendency of AuNCs in water*. The PL origin of the AuNCs is the Au(I)-SG oligomers on their surface, whose structure is shown in Figure S1a. One moiety, $Au(I)_2$-SG, from the oligomeric molecule is defined as the emissive unit for the computational study (Figure S8a).[1,8] In the emissive unit, the ligand, glutathione, acts as the electron donor, and the gold atoms as the acceptor.[7] PL occurs when the photon-excited electrons transfer from the ligand to gold, which is a typical intramolecular charge transfer (ICT) process, since the Au(I)-SG oligomer is considered as a whole molecule. Twisting of the ligand might happen during ICT, namely TICT, which can decrease the PLQY and photostability of fluorophores.[9] The tendency of TICT in the emissive unit is studied by DFT and TD-DFT calculations. This is to confirm if TICT can take place in the AuNCs and affect their PL properties.

The minima potential energy profile of the emissive unit in water is constructed as a function of the dihedral angle of Au(I)-S-$CH_2$-CH. The dihedral angles are fixed at various values, while the rest parts of the emissive unit are subject to the geometry optimization. Energy potentials of ground and excited states of the emissive unit are calculated at every 10 degrees of rotation, which are shown in Table S1. The results indicate that, upon excitation, the donor part is highly prone to be twisted (rotation) by 50 degrees in water, resulting in a TICT process. Note that one AuNC particle possesses tens of the twisting ligands. The TICT affect is immense as a result of collective effects. It explains discrete AuNCs in water suffer from low PL efficiency and photostability.

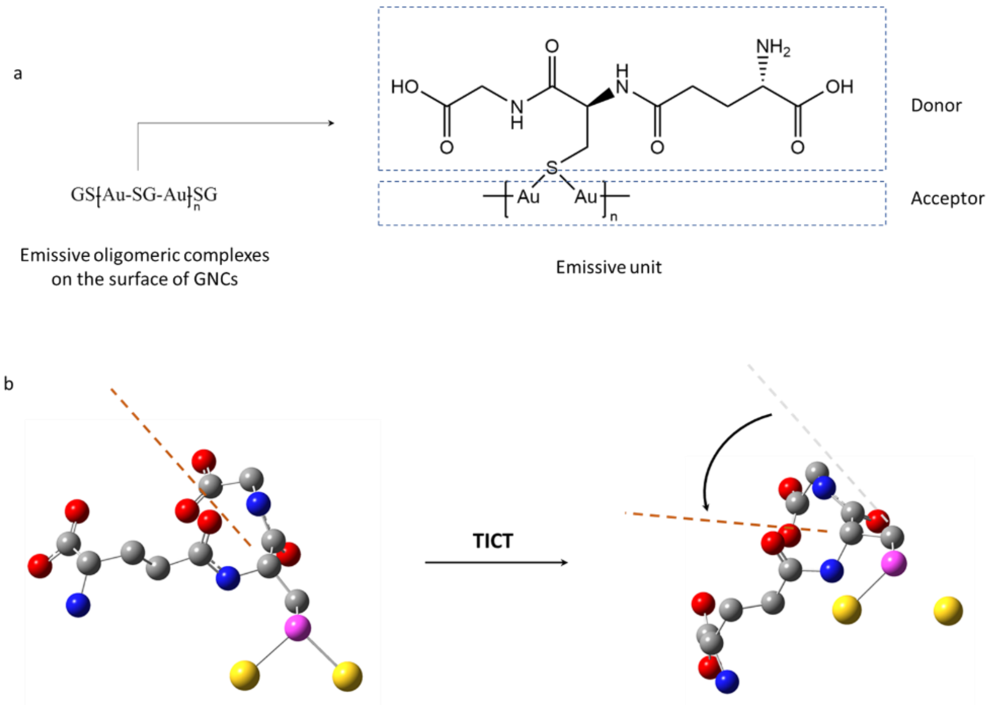


**Figure S7.** (a) The emissive unit of the AuNCs. (b) Calculated TICT process. The glutathione ligand is highly prone to rotation in water upon excitation. Au (yellow), S (purple), C (grey), O (red) are shown in the optimized structures.

**Table S1.** The energy potentials of the ground and excited states, as a function of the dihedral angle of Au(I)-S-$CH_2$-CH in one emissive unit in the discrete AuNCs.

| Twisted angle (º) | Relative ground state energy potential (eV) | Relative excited state potential (eV) |
|---|---|---|
| 0 | 0 | 2.72409 |
| -10 | 0.00141 | 2.73392 |
| -20 | 0.00819 | 2.72066 |
| -30 | 0.02003 | 2.65043 |
| -40 | 0.03897 | 2.60779 |
| -50 | 0.06084 | 2.60445 |

*TICT tendency of AuNCs in the supraparticle structure*. The tendency of TICT is further studied when AuNCs are clustered into supraparticles, in which the nanoclusters are non-covalently bonded. Two emissive units from different AuNCs are employed and two pair of Au(I) atoms are separated at a fixed distance of 9.2 Å, which is measured from the HR-TEM analysis (Figure S3). After the structural optimization and frequency check, single point calculations are carried out on each optimized structure, which are shown in Table S2. The results show that TICT is located at a higher energy potential, toward which the LE state has to overcome a high energy barrier. It means that the TICT process is extremely unfavorable for AuNCs in the supraparticle structure. More energy of the electron transition is converted into PL emission because of the TICT inhibition.

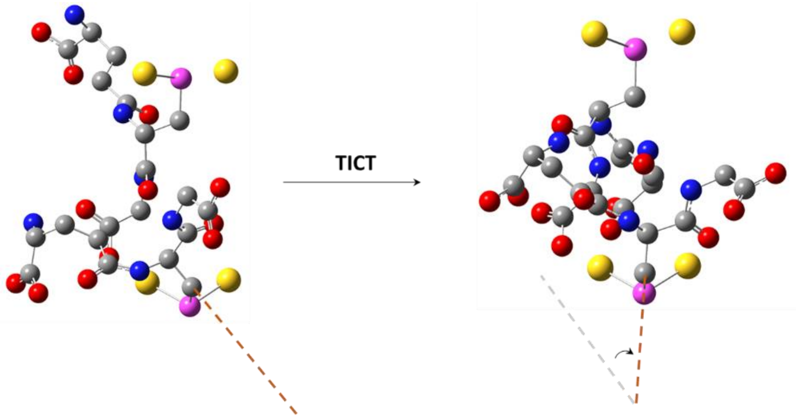


**Figure S8.** Calculated TICT process of one emissive unit in a supraparticle. The twisting of the glutathione ligand is extremely unfavorable upon excitation. Au (yellow), S (purple), C (grey), O (red) are shown in the optimized structures.

**Table S2.** The energy potentials of the ground and excited states, as a function of the dihedral angle of Au(I)-S-$CH_2$-CH in one emissive unit when supraparticles are formed.

| Twisted angle (º) | Relative ground state energy potential (eV) | Relative excited state potential (eV) |
|---|---|---|
| 0 | 0 | 2.72086 |
| -10 | 0.00936 | 2.72619 |
| -20 | 0.76534 | 3.48657 |
| -30 | 0.74022 | 3.46486 |
| -40 | 1.12199 | 3.77925 |
| -50 | 1.11519 | 3.77286 |

## S6. The effect of water on the PL properties of AuNC-SPs

*Addition of water into the AuNC-SP system*. The presence of water in AuNC-SPs facilitates TICT. Figure S9 reveals how the PL intensity and peak wavelength of AuNC-SPs change with the addition of water. A small amount water can decrease the PL intensity significantly. As the volume ratio of water versus dodecane reaches 4.5%, the PL intensity decreases by 92%, indicating a remarkable quenching effect by water. Meanwhile, the peak wavelength red shifts from 586 to 599 nm, suggesting an enhanced TICT effect. All results reveal that water softens the supraparticle structure, facilitating TICT and quenching the emission intensity profoundly.

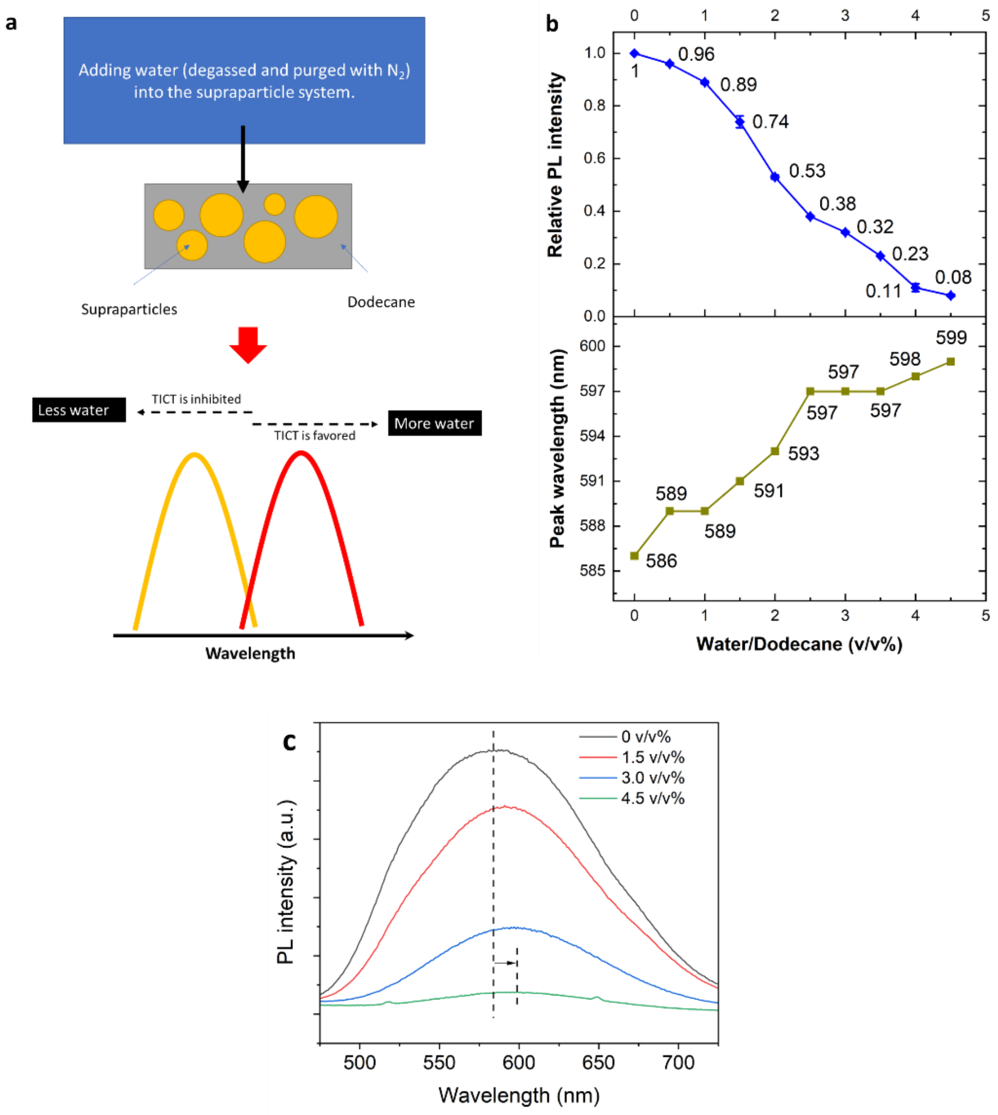


**Figure S9**. Study on the effect of the water amount on the PL properties of AuNC-SPs. (a) Schematic illustration: the addition of water (oxygen-free) into the AuNC-SP system, which facilitates TICT and redshifts the emission peak. (b) The relative emission intensity and peak wavelength of the AuNC-SP system as a function of the volume ratio of water versus dodecane. (c) The PL spectra of AuNC-SPs upon the addition of water at different volume ratios versus dodecane (v/v%).

*Absorption and extraction of water in the AuNC-SP system*. The AuNC-SPs are sensitive to water, tending to absorb water in the water-rich atmosphere. As shown in Figure S10, the supraparticle system absorbs water from the water-saturated $N_2$ atmosphere, as reflected by the greatly decreased PL intensity (100%→55%) and the red-shifted peak wavelength (586 nm→593 nm), namely an increased TICT effect. The TICT effect is not further enhanced once the supraparticle system absorbs enough water from the ambient environment (after 80 min).

On the other hand, the supraparticle system losses water molecules when there is little water in the ambient environment. As shown in Figure S11, the supraparticle system keeps releasing water in the vacuum, as reflected by the constantly decreased peak wavelength (586 nm→583 nm), namely a decreased TICT effect. Interestingly, the PL intensity increases at first because of stronger TICT inhibition (100%→105%), but decreases gradually as more water is removed (105%→80%), despite an even enhanced TICT inhibition. These results suggest that a trace amount of water facilitates the emission enhancement. This phenomenon is commonly observed in the fluorescent perovskite

nanocrystals, whose PL intensity can be easily quenched by water. But a slight mount of water can passivate the surface trap states and thereby increase the PLQY of perovskite nanocrystals.[10] Water may play a similar role in the supraparticles as it does in the perovskite nanocrystals. It is clear that a trace amount of water can help the recombination of the excitons go through a radiative pathway. Systematic investigation is required to shed light on its mechanism in the future.

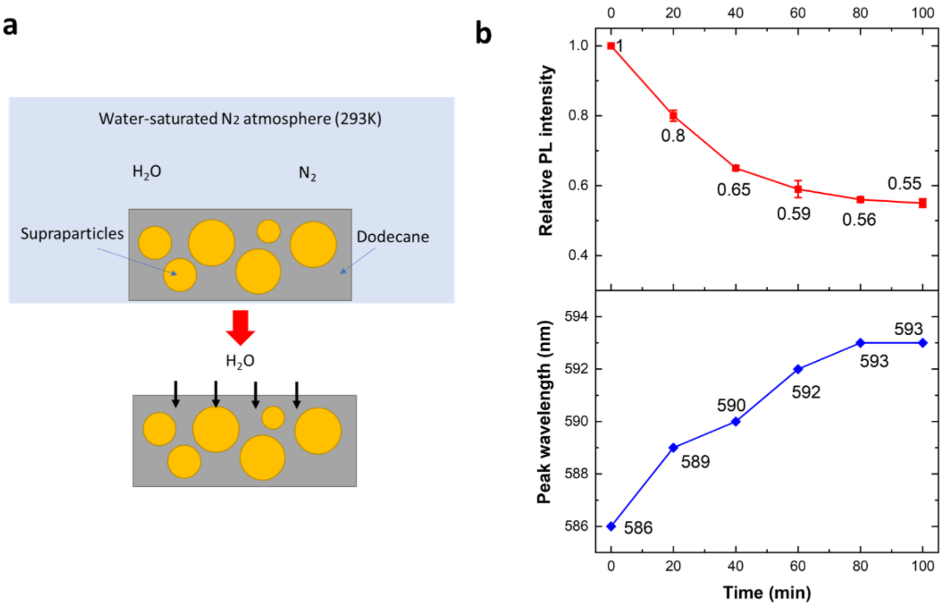


**Figure S10**. (a) Schematic illustration: the AuNC-SP system is put in the water-saturated $N_2$ atmosphere to make the supraparticle system absorb water. (b) The relative emission intensity and peak wavelength of the AuNC-SP system as a function of time in the water-saturated $N_2$ atmosphere.

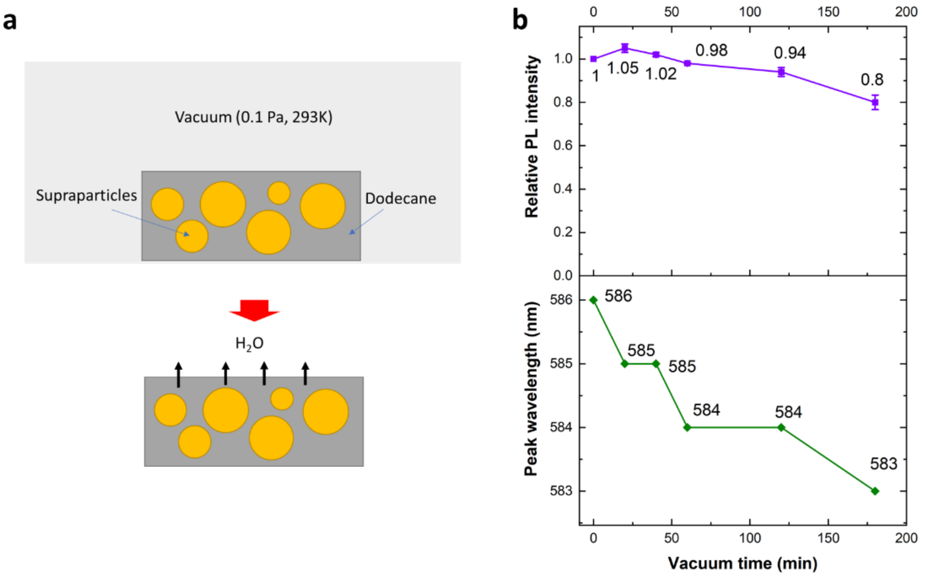


**Figure S11**. (a) Schematic illustration: the AuNC-SP system is put under vacuum, making the system release water molecules constantly. (b) The relative emission intensity and peak wavelength of the AuNC-SP system as a function of time in the vacuum (0.1 Pa, 293 K). Before each measurement, the samples are always purged with dry nitrogen gas and added enough degassed and water-free dodecane to compensate the loss of dodecane in the vacuum.

*Over-evaporation during the formation of AuNC-SPs*. Over-evaporation in the synthetic process can lower the PLQY of the as-prepared supraparticles, further demonstrating the favorable effect of a trace

amount of water in the supraparticles. Figure S12 illustrates how the AuNC-SPs are formed during the evaporation of the reverse micelles. The PL intensity decreases gradually with the increase in heating time after reaching its maximum value. whilst, the emission peak wavelength keeps decreasing, indicating a constant removal of water. Interestingly, Figures S12c and S12d reveal that over-removal of water leads to the interconnection/mergence of the supraparticles. Therefore, a trace amount of water can stabilize the supraparticle structure. The structural disruption could decrease the PLQY (Figure S12b).

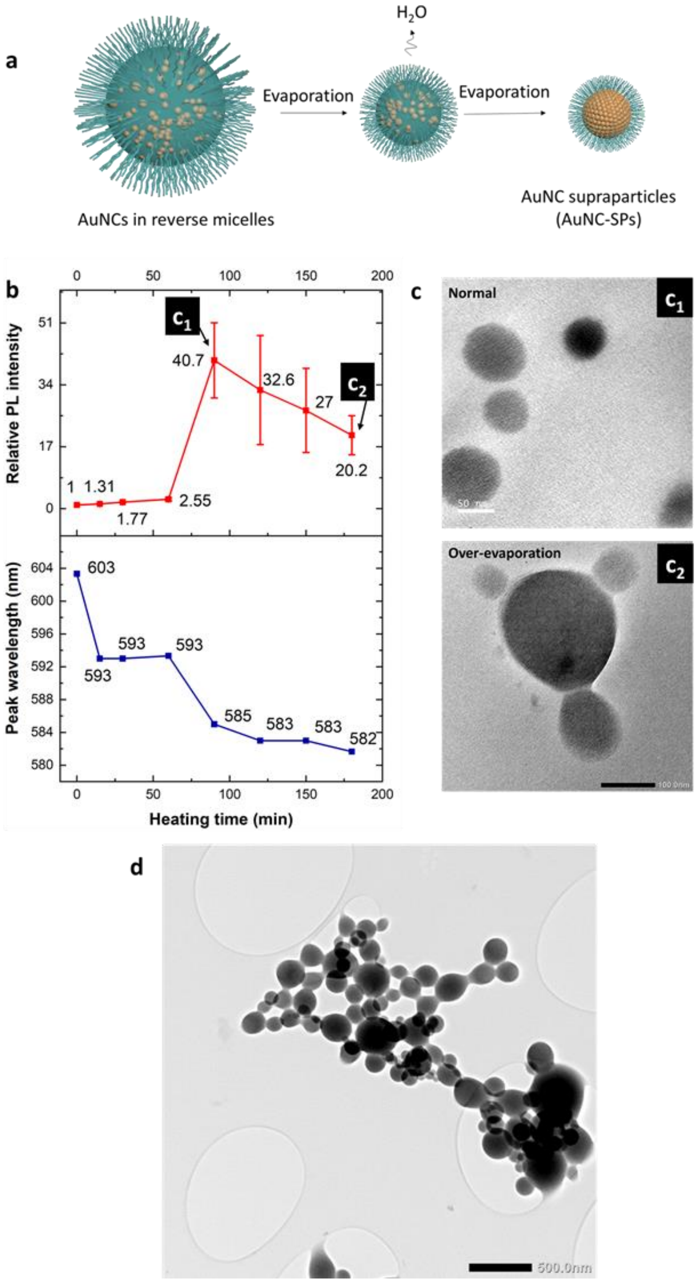


**Figure S12**. (a) Schematic illustration: the synthesis of AuNC-SPs from reverse micelles that are heated at 80 °C. (b) Evolutions of the relative emission intensity (up) and emission peak wavelength (down) during the formation process of AuNC-SPs. (c) STEM images of AuNC-SPs in two different stages: normal AuNC-SPs that have the highest PL intensity ($c_1$) and over-evaporated AuNC-SPs ($c_2$). (d) STEM image: an overview of interconnected supraparticles after over-evaporation.

Generally, complete removal of water is detrimental to the PL performance of supraparticles and their structural stability. Thus, water cannot be removed totally. In fact, there is always an equilibrium

between the water in the supraparticles and that in the ambient environment. Their chemical potentials should be equivalent:

$$\mu_{water}\,(supraparticles) = \mu_{water}(ambient)$$

When $\mu_{water}\,(supraparticles) < \mu_{water}(ambient)$, the supraparticle system absorbs the ambient water, favoring TICT and increasing the emission wavelength; when $\mu_{water}\,(supraparticles) > \mu_{water}(ambient)$, the supraparticle system transfers the water to the ambient environment, inhibiting TICT and increasing the emission wavelength. Most but not all of water, should be removed from the supraparticles for an optimized PL performance.

## S7. The PL kinetics in the AuNCs

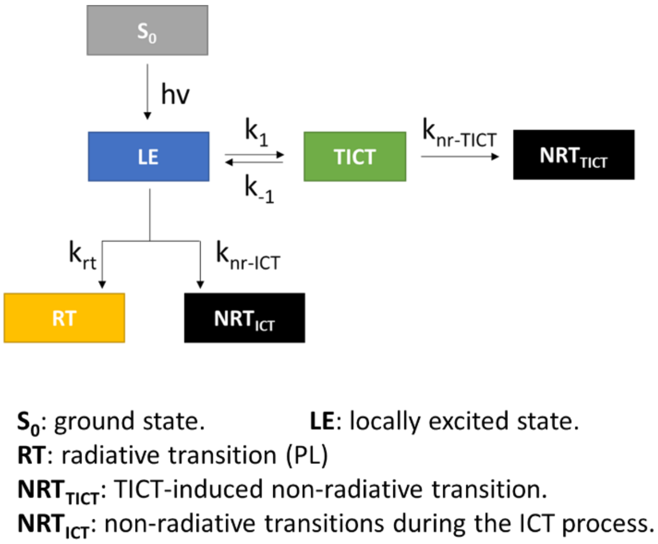


**Figure S13**. Schematic illustration: the kinetic process of PL in the AuNCs.

Upon absorption of photons, locally excited (LE) states are generated from the ground state ($S_0$):

$$S_0 \xrightarrow{hv} LE$$

Transitions of the LE states proceed in terms of intramolecular charge transfer (ICT) process, including both radiative transition (NR, or PL) and non-radiative transition (NRT).

$$LE \xrightarrow{k_{rt}} RT\ (PL)$$

$$LE \xrightarrow{k_{nr-ICT}} NRT_{ICT}$$

Under some conditions, TICT can take place, resulting in a non-radiative electron transition.

$$LE \xrightarrow{k_1} TICT$$

$$TICT \xrightarrow{k_{-1}} LE$$

$$TICT \xrightarrow{k_{nr-TICT}} NRT_{TICT}$$

Based on the mechanism above, the rates of RT and NRT can be expressed as follows:

$$\frac{d[RT]}{dt} = K_r[LE] = k_{rt}[LE]$$

$$\frac{d[NRT]}{dt} = K_{nr}[LE] = k_{nr-ICT}[LE] + k_{nr-TICT}[TICT]$$

Since the TICT state is a transition state, its amount remains unchanged with the time, based on the steady-state approximation:

$$\frac{d[TICT]}{dt} = k_1[LE] - k_{-1}[TICT] - k_{nrt-TICT}[TICT] = 0$$

Correlating the equations, the rate coefficients $K_r$ and $K_{nr}$ can be obtained:

$$K_r = k_{rt}$$

$$K_{nr} = k_{nr-ICT} + \frac{k_1 k_{nrt-TICT}}{k_{-1} + k_{nrt-TICT}}$$

The PL efficiency of AuNCs, namely PLQY($Q_r$), is the radiative proportion of the electron transition:

$$Q_r = \frac{\frac{d[RT]}{dt}}{\frac{d[RT]}{dt} + \frac{d[NRT]}{dt}} = \frac{K_r}{K_r + K_{nr}}$$

Substituting $K_r$ and $K_{nr}$,

$$Q_r = \frac{k_{rt}}{k_{rt} + k_{nr-ICT} + \frac{k_1 k_{nrt-TICT}}{k_{-1} + k_{nrt-TICT}}}$$

Clearly, PLQY of the AuNCs can be easily changed by varying the values of $K_r$ and $K_{nr}$. Maximizing $K_r$, and minimizing $K_{nr}$ by restricting TICT and other thermal motions like particle movement and ligand fluctuation,[11,12] can result in a significant increase in the PL efficiency.

## S8. PL enhancement of AuNCs in different matrices

*PL enhancement of AuNCs in the polymer film*. Figure S14a shows the PL spectra of AuNCs in the polymer film at 293 K. AuNCs give out a stronger emission (an approximately 4-fold intensity increase) in the PVA film than in water. The emission band is centered at 589 nm, while that of aqueous AuNCs is centered at ~602 nm. The polymer matrix provides a more rigid medium than water does to the AuNCs, in which TICT and other thermal motions are not favored. It means that the overall rate coefficient of non-radiative transition is decreased, resulting in the emission enhancement.

*PL enhancement of AuNCs in the frozen ice*. Figure S14b shows the PL spectra of AuNCs in aqueous solution at 293 K and 77 K. The PL intensity rises intensively with the decrease in temperature. At 77 K, AuNCs emit the strongest emission (an approximately 9-fold intensity increase) with the peak wavelength at ~585 nm, compared to the weak emission at 293 K, with a peak at ~602 nm. Likely in the PVA matrix, TICT and other thermal motions, are restricted in the extremely frozen medium. But the restriction effect is stronger due to a lower temperature and a more rigid medium.

*PL enhancement of AuNCs in the superaparticle structure*. AuNC-SPs exhibit a blue-shifted emission with respect to that of AuNCs, whereas their excitation spectra are almost identical (Figure 2f). The emission peak shifts from ~602 nm to ~585 nm when the supraparticles are generated. In addition, the emission peak wavelength of AuNC-SPs at 293 K is almost the same with that of AuNCs at 77 K (Figure S14c). The results clearly indicate that the TICT process has been greatly inhibited in AuNC-SPs without lowering the temperature. The synergistic effect of the closely packed nanoclusters and the

slight amount of water throughout the structure has resulted in the greatest emission enhancement (a 54-fold intensity increase) for AuNCs in the superaparticles.

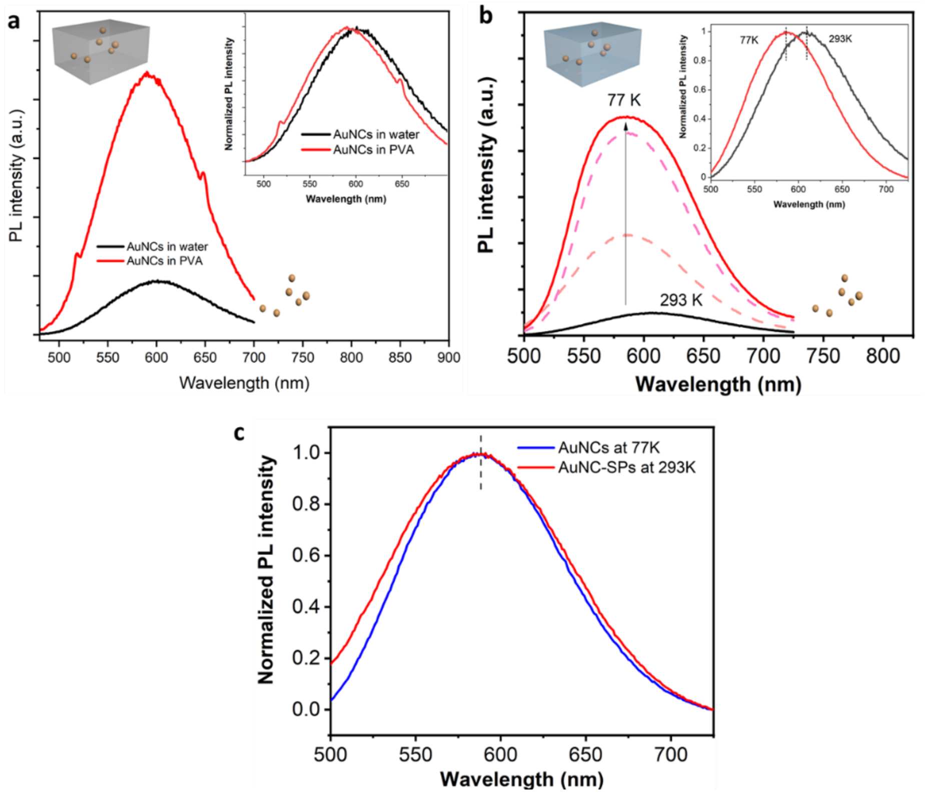


**Figure S14.** (a) Emission spectra of AuNCs in water and in the PVA film. Inset: the normalized PL spectra in water and the PVA film. (b) Emission spectra of AuNCs at 293 K and 77 K. Inset: the normalized PL spectra at these two temperatures. (c) Normalized PL spectra of AuNCs at 77 K and AuNC-SPs at 293 K. Their emission bands are centered at the same wavelength position.

## S9. Properties of other MNCs and MNC-SPs (M=Au, Ag, and Cu)

Properties of GSH-stabilized AgNCs, CuNCs, as well as histidine-stabilized AuNCs (namely His-AuNCs) have been all well-studied in the previous research.[2–4]

*Performance enhancement in the supraparticles of AgNCs and CuNCs*. The PL property of AgNCs originates from the Ag-SG complexes on the surface.[2] The AgNCs show aggregation-induced emission enhancement (AIEE) effect when the particles are clustered. As for the CuNCs, the Cu-SG complexes ($CuSG_2$) are believed responsible for the PL emission, which also shows AIEE affect.[3] Therefore, both the AgNC and CuNCs share similar PL mechanism with the GSH-stabilized AuNCs that are discussed above. It means that charge transfer between the ligand and metal gives rise to the PL properties. Formation of AgNC-SPs and CuNC-SPs results in the presence of strong emissions at ~600 nm (Figures S15a and S15b), which are greatly quenched when the nanoclusters are dispersed in water, largely due to the TICT effect. Lowering the temperatures of the aqueous dispersions of AgNC and CuNCs can greatly increase the PL intensity (Figures S16a and S16b). As discussed above, TICT and other thermal motions are impeded by the frozen ice, giving rise to the emission enhancement.

*Performance enhancement in the supraparticles of His-AuNCs*. PL properties of His-AuNCs are reported to be originated from the gold kernel,[4,13] which is different from the GSH-stabilized AuNCs studied in this work.[7,11] The emission is less associated with the ligand-metal charge transfer, thus TICT is unlikely to affect the PLQY of His-AuNCs significantly. Formation of His-AuNC-SPs can still lead to an emission enhancement, due to the restriction of the thermal motions that can consume the energy of electron transitions. The PL intensity of His-AuNCs is observed at temperature of 77 K

(Figure S16c), demonstrating the effect of motion inhibition. However, the PL efficiency increase in His-AuNC-SPs is not as much as that in AgNC-SPs or CuNC-SPs, largely because TICT is not involved in the PL emission of His-AuNCs. The emission band of His-AuNC-SPs is broadened compared to that of His-AuNCs (Figure S15c), making the blue emission more greenish, which is probably ascribed to strengthened electron-electron interactions and electron-surface/defect scattering effect.[14]

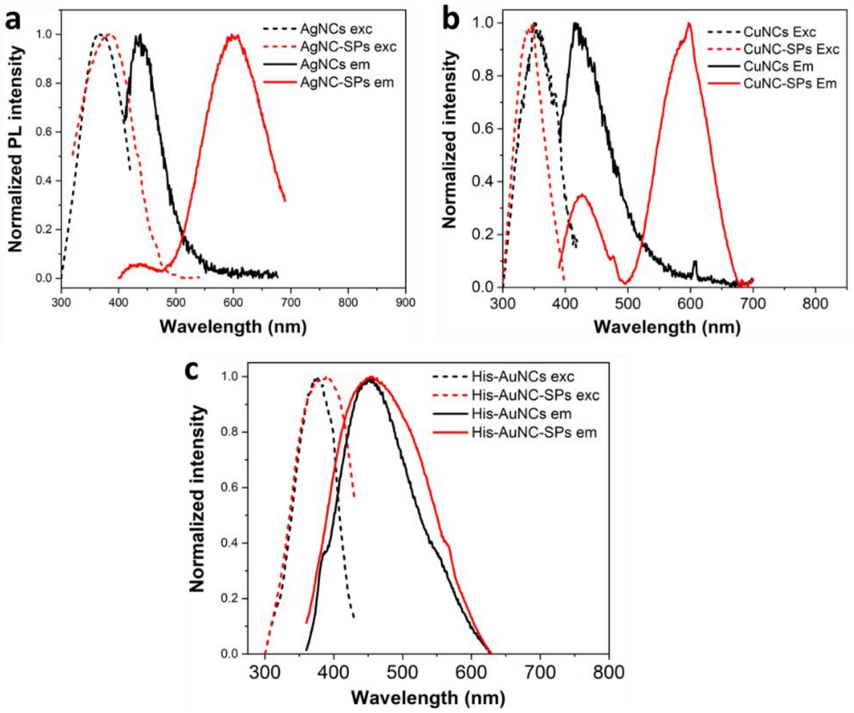


**Figure S15.** Normalized excitation (dash line) and emission (solid line) spectra of AgNC (a), CuNCs (b), His-AuNCs (c) and their corresponding supraparticles.

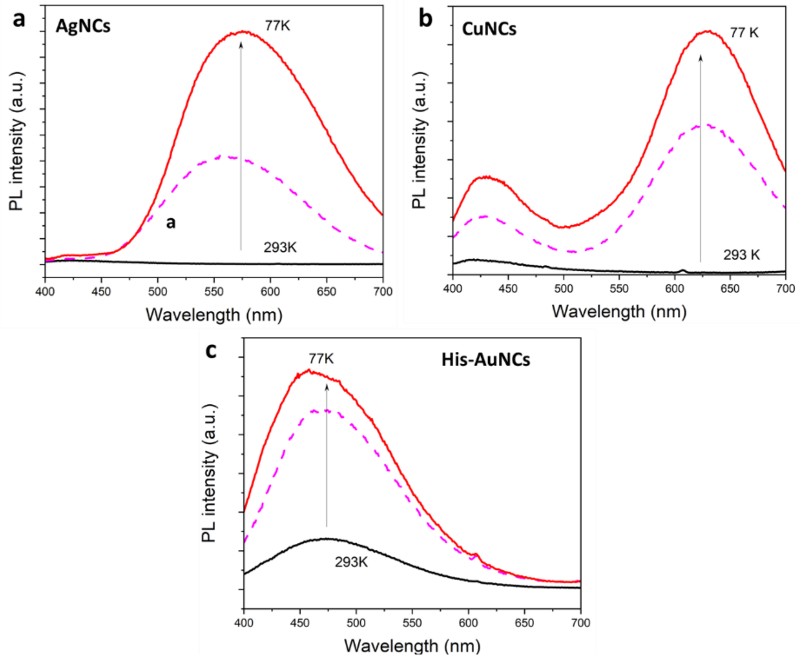


**Figure S16.** PL emission spectra of AgNC, CuNCs and His-AuNCs aqueous solutions under different temperatures. All PL intensities at 77 K are higher than those at 293 K.

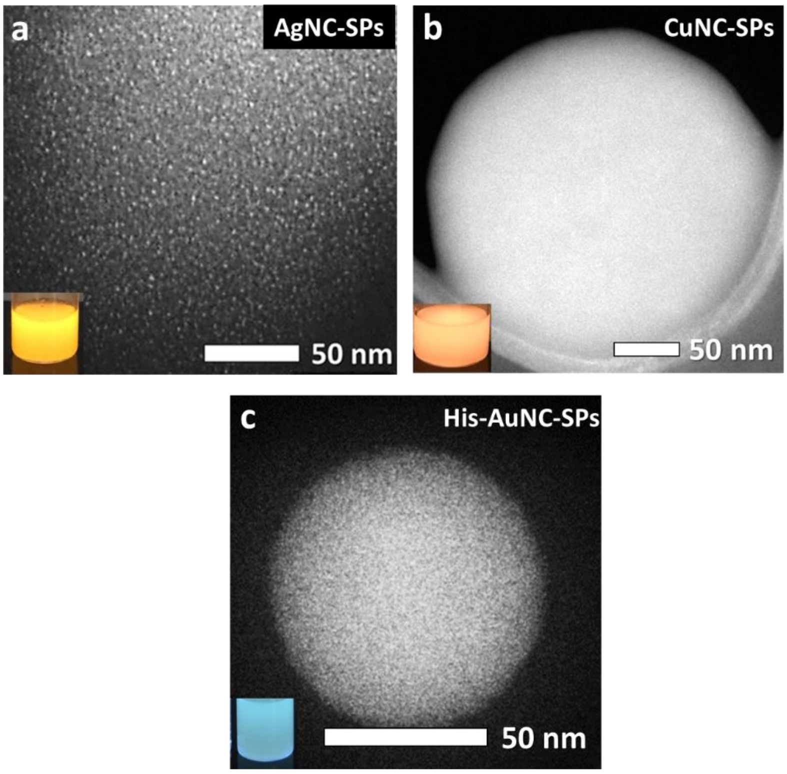


**Figure S17.** Morphologies of MNC-SPs. Dark-field STEM images of AgNC-SPs (a), CuNC-SPs (b) and His-AuNC-SPs (c).

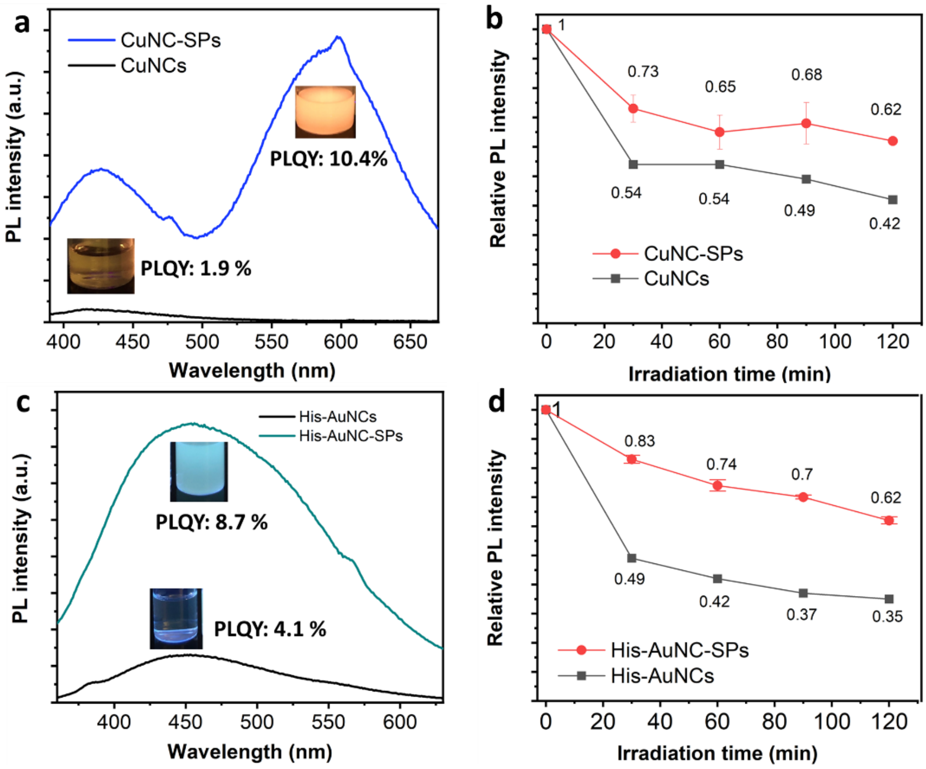


**Figure S18.** (a) PL spectra of CuNCs and CuNC-SPs. (b) Relative PL intensity evolution of CuNCs and CuNC-SPs under continuous UV irradiation at 365 nm, 5.3 mW/cm$^2$. (c) PL spectra of AgNCs and AgNC-SPs. (b) Relative PL intensity evolution of His-AuNCs and His-AuNC-SPs under continuous UV irradiation at 365 nm, 5.3 mW/cm$^2$.

*The effect of water in the PL properties of AgNC-SPs and CuNC-SPs*. Similar to AuNC-SPs, the TICT effect caused by water is also appliable to AgNC-SPs and CuNC-SPs. Figures S19 and S20 show how the PL property of AgNC-SPs and CuNC-SPs changes both in the water-rich $N_2$ atmosphere and under vacuum, respectively. Similar to AuNC-SPs, the absorption of water red-shifts the emission peak and decreases the PL intensities of AgNC-SPs and CuNC-SPs, indicating an increased TICT effect; while excessive removal of water blue shifts the emission peak and decreases the PL intensity because a trace amount of water in the supraparticles can contribute to the emission enhancement. The results suggest water plays a similar role in the PL properties of AuNC-SPs, AgNC-SPs and CuNC-SPs. Notably, AgNC-SPs and CuNC-SPs are more sensitive to water than their gold counterparts, as reflected by the greater emission changes upon water absorption.

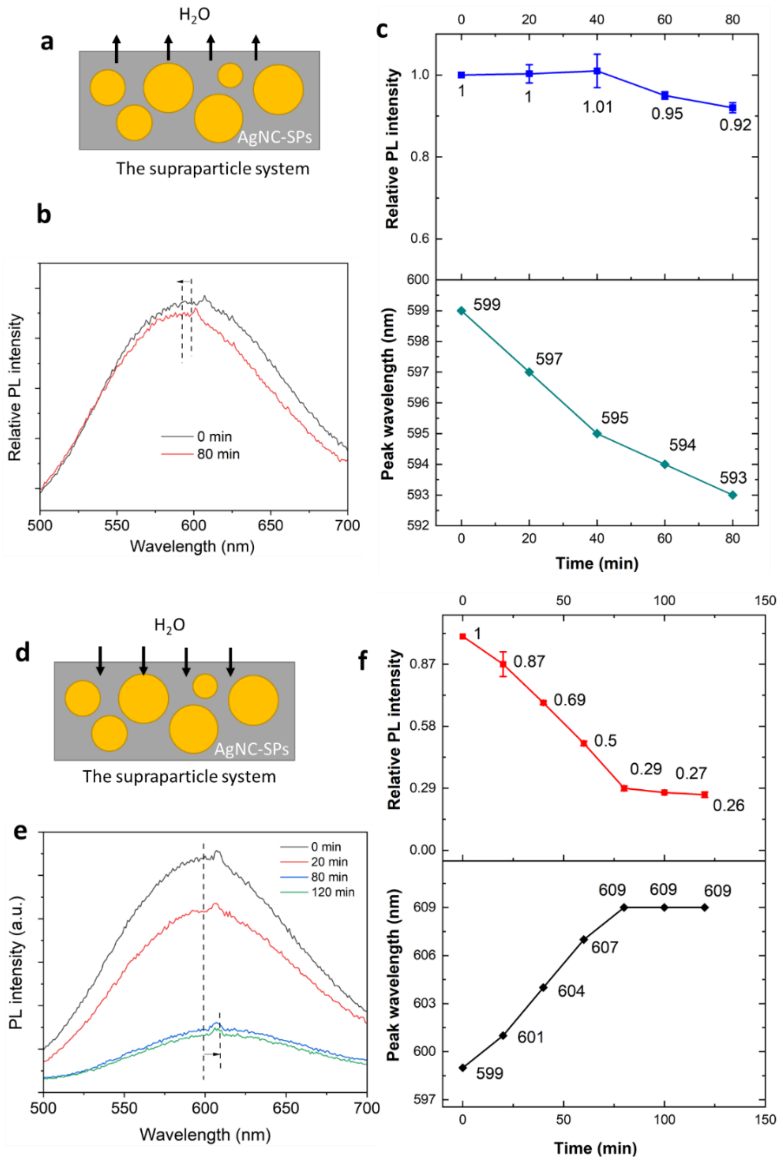


**Figure S19**. Water removal and absorption of AgNC-SPs and their PL properties. (a) Water removal of the AgNC-SP system under vacuum (0.1 Pa, 293 K). (b) PL spectra of AgNC-SPs after water removal at different time intervals. (c) The emission intensity and peak wavelength evolutions of AgNC-SPs during water removal. (d) Water absorption by the AgNC-SP system in the water-saturated $N_2$ atmosphere (293 K). (e) PL spectra of AgNC-SPs after water absorption at different time intervals. (f) The emission intensity and peak wavelength evolutions of AgNC-SPs during water absorption.

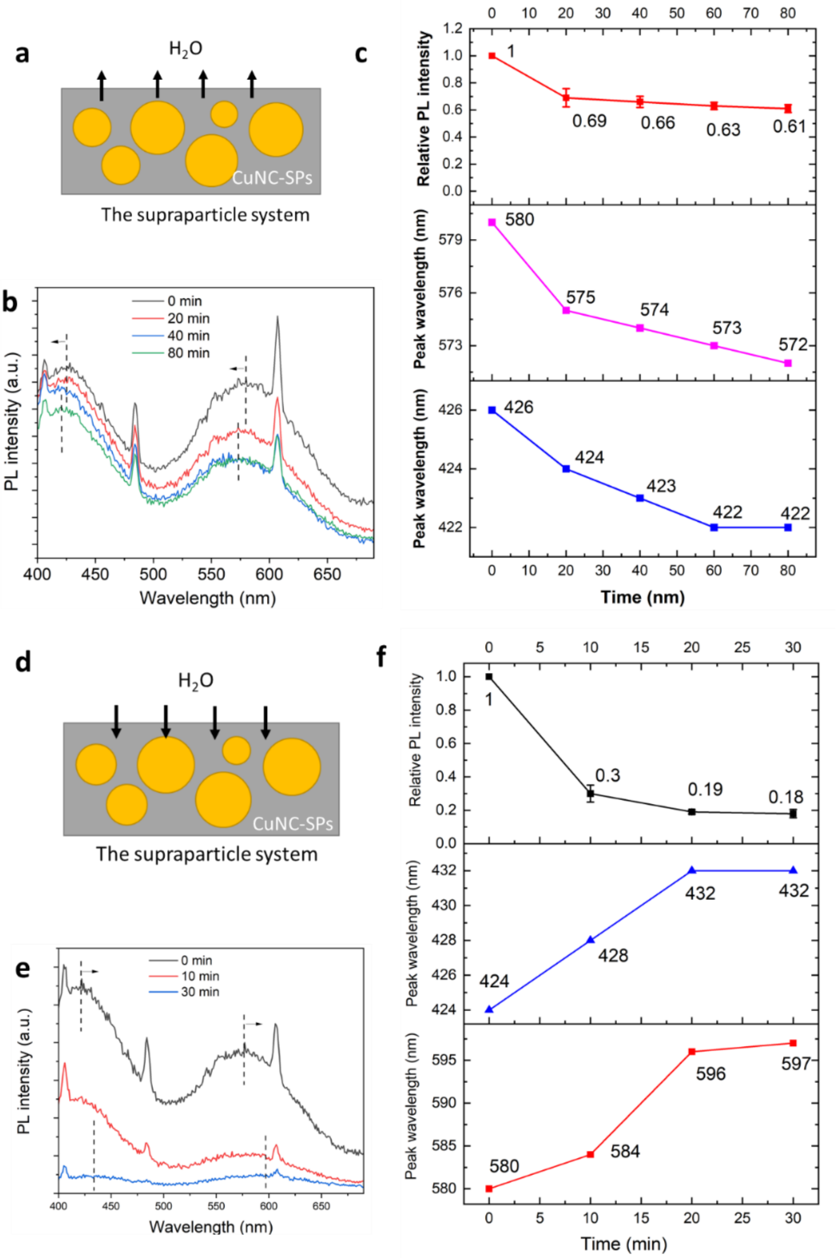


**Figure S20**. Water removal and absorption of CuNC-SPs and their PL properties. (a) Water removal of the CuNC-SP system under vacuum (0.1 Pa, 293 K). (b) PL spectra of CuNC-SPs after water removal at different time intervals. (c) The emission intensity and peak wavelength evolution of CuNC-SPs during water removal. (d) Water absorption by the CuNC-SP system in the water-saturated $N_2$ atmosphere (293 K). (e) PL spectra of CuNC-SPs after water absorption at different time intervals. (f) The emission intensity and peak wavelength evolutions of CuNC-SPs during water absorption.

# References


[1] Z. Luo, X. Yuan, Y. Yu, Q. Zhang, D. T. Leong, J. Y. Lee, J. Xie, *J. Am. Chem. Soc.* **2012**, *134*, 16662–16670.
[2] K. Zheng, X. Yuan, K. Kuah, Z. Luo, Q. Yao, Q. Zhang, J. Xie, *Chem. Commun.* **2015**, *51*, 15165–15168.
[3] X. Jia, J. Li, E. Wang, *Small* **2013**, *9*, 3873–3879.
[4] X. Yang, M. Shi, R. Zhou, X. Chen, H. Chen, *Nanoscale* **2011**, *3*, 2596.
[5] F. Weigend, R. Ahlrichs, *Phys. Chem. Chem. Phys.* **2005**, *7*, 3297–3305.
[6] P. N. Day, R. Pachter, K. A. Nguyen, R. Jin, *J. Phys. Chem. A* **2019**, *123*, 6472–6481.
[7] Z. Wu, R. Jin, *Nano Lett.* **2010**, *10*, 2568–2573.
[8] J. Akola, M. Walter, R. L. Whetten, H. Häkkinen, H. Grönbeck, *J. Am. Chem. Soc.* **2008**, *130*, 3756–3757.

[9] X. Liu, Q. Qiao, W. Tian, W. Liu, J. Chen, M. J. Lang, Z. Xu, *J. Am. Chem. Soc.* **2016**, *138*, 6960–6963.
[10] H. Moon, C. Lee, W. Lee, J. Kim, H. Chae, *Adv. Mater.* **2019**, *31*, 1804294.
[11] K. Pyo, V. D. Thanthirige, K. Kwak, P. Pandurangan, G. Ramakrishna, D. Lee, *J. Am. Chem. Soc.* **2015**, *137*, 8244–8250.
[12] Q. Li, M. Zhou, W. Y. So, J. Huang, M. Li, D. R. Kauffman, M. Cotlet, T. Higaki, L. A. Peteanu, Z. Shao, R. Jin, *J. Am. Chem. Soc.* **2019**, *141*, 5314–5325.
[13] J. Zheng, C. Zhang, R. M. Dickson, *Phys. Rev. Lett.* **2004**, *93*, 077402.
[14] P. Yu, X. Wen, Y.-R. Toh, J. Tang, *J. Phys. Chem. C* **2012**, *116*, 6567–6571.
